# Disparities in COVID-19 related outcomes in the United States by race and ethnicity pre-vaccination era: an umbrella review of meta-analyses

**DOI:** 10.3389/fpubh.2023.1206988

**Published:** 2023-09-07

**Authors:** Khanh N. C. Duong, Lan M. Le, Sajesh K. Veettil, Pantakarn Saidoung, Warintorn Wannaadisai, Richard E. Nelson, Mike Friedrichs, Barbara E. Jones, Andrew T. Pavia, Makoto M. Jones, Matthew H. Samore, Nathorn Chaiyakunapruk

**Affiliations:** ^1^Department of Pharmacotherapy, College of Pharmacy, University of Utah, Salt Lake City, UT, United States; ^2^International Medical University, School of Pharmacy, Department of Pharmacy Practice, Kuala Lumpur, Malaysia; ^3^Faculty of Pharmacy, Mahidol University, Bangkok, Thailand; ^4^Division of Epidemiology, School of Medicine, University of Utah, Salt Lake City, UT, United States; ^5^IDEAS Center, Veterans Affairs Salt Lake City Health Care System, Salt Lake City, UT, United States; ^6^Utah Department of Health, Salt Lake City, UT, United States; ^7^Division of Pulmonary & Critical Care, University of Utah, Salt Lake City, UT, United States; ^8^Division of Pediatric Infectious Diseases, University of Utah, Salt Lake City, UT, United States

**Keywords:** COVID-19, race/ethnicity, health equity, the US, umbrella review

## Abstract

**Background:**

Meta-analyses have investigated associations between race and ethnicity and COVID-19 outcomes. However, there is uncertainty about these associations’ existence, magnitude, and level of evidence. We, therefore, aimed to synthesize, quantify, and grade the strength of evidence of race and ethnicity and COVID-19 outcomes in the US.

**Methods:**

In this umbrella review, we searched four databases (Pubmed, Embase, the Cochrane Database of Systematic Reviews, and Epistemonikos) from database inception to April 2022. The methodological quality of each meta-analysis was assessed using the Assessment of Multiple Systematic Reviews, version 2 (AMSTAR-2). The strength of evidence of the associations between race and ethnicity with outcomes was ranked according to established criteria as convincing, highly suggestive, suggestive, weak, or non-significant. The study protocol was registered with PROSPERO, CRD42022336805.

**Results:**

Of 880 records screened, we selected seven meta-analyses for evidence synthesis, with 42 associations examined. Overall, 10 of 42 associations were statistically significant (*p* ≤ 0.05). Two associations were highly suggestive, two were suggestive, and two were weak, whereas the remaining 32 associations were non-significant. The risk of COVID-19 infection was higher in Black individuals compared to White individuals (risk ratio, 2.08, 95% Confidence Interval (CI), 1.60–2.71), which was supported by highly suggestive evidence; with the conservative estimates from the sensitivity analyses, this association remained suggestive. Among those infected with COVID-19, Hispanic individuals had a higher risk of COVID-19 hospitalization than non-Hispanic White individuals (odds ratio, 2.08, 95% CI, 1.60–2.70) with highly suggestive evidence which remained after sensitivity analyses.

**Conclusion:**

Individuals of Black and Hispanic groups had a higher risk of COVID-19 infection and hospitalization compared to their White counterparts. These associations of race and ethnicity and COVID-19 outcomes existed more obviously in the pre-hospitalization stage. More consideration should be given in this stage for addressing health inequity.

## 1. Introduction

As of June 7, 2023, more than 760 million people worldwide had documented COVID-19 infections. Approximately 6.9 million lost their lives to COVID-19 (1). Notably, the United States (US) accounted for more than 103 million confirmed cases and more than 1.1 million deaths (1). A large number of studies, including several meta-analyses, have reported on disparities in COVID-19 health outcomes across racial and ethnic population groups (2). Health outcomes have been defined in various ways, including rates of infection, hospitalization, and death. As reported by the Centers for Disease Control and Prevention (CDC), age-adjusted COVID-19 mortality rates have been higher for Black non-Hispanic and Hispanic populations than for other groups (3). However, disparities of other types of COVID-19 outcomes have not been consistently observed.

Several systematic reviews and meta-analyses (4–7) also have investigated associations between race and ethnicity and COVID-19-related outcomes in various population groups ranging from the general population to hospitalized patients with COVID-19. Some studies reported that ethnic minorities had a higher mortality rate than White individuals (4, 5, 8). However, no significant associations between COVID-19 death among those who were infected and those who got hospitalized were revealed in another study (8). Our goal in this paper was to synthesize data on disparities, using the umbrella review method to systematically summarize and quantify all relevant meta-analyses of associations between race and ethnicity and COVID-19-related outcomes in the US. We categorized studies based on the outcome and population at risk, whether pre-hospitalization or post-hospitalization. We summarized and quantified evidence from multiple meta-analyses on the same outcomes, to assess the quality and strength of the associations and grade the level of evidence (9–11). Our review was performed to gain insight into the intersection between infectious disease dynamics and health outcomes, thereby uncovering the role of underlying social and structural determinants of health. Measurement of racial and ethnic disparities is a key step toward developing and implementing strategies for their amelioration.

## 2. Method

### 2.1. Search strategy and selection criteria

This systematic review of meta-analyses followed the 2020 Preferred Reporting Items for Systematic Reviews and Meta-analyses (PRISMA) reporting guideline (12) and the Meta-analysis of Observational Studies in Epidemiology (MOOSE) reporting guideline (13). A comprehensive literature search was performed in PubMed, Embase, the Cochrane Database of Systematic Reviews (CDSR), and Epistemonikos from the database inception to April 25, 2022. Our search strategies combined search terms related to COVID-19, race and ethnicity, health inequity/disparity, and systematic review/meta-analysis. We also manually searched the cited references of the selected articles and reviews. There were no restrictions on time and language in searching (Supplementary Data). Two authors (LL and KD) independently screened titles or abstracts and examined the full text of potentially eligible articles. Discrepancies were resolved by a third reviewer (SV).

We included meta-analyses of observational studies conducted on the US population investigating COVID-19-related outcomes by race and ethnicity. Outcomes included COVID-19 infection, hospitalization, Intensive care unit (ICU) admission, and other outcomes as defined by the original authors. When more than one meta-analysis on the same association was available, we selected the association with the largest dataset and effect size-adjusted with a comprehensive set of confounding variables, as previously described (14–17). We excluded meta-analyses that provided insufficient data for quantitative synthesis. Detailed eligibility criteria and a description of the selection between overlapping meta-analyses are provided in Supplementary Table S1.

### 2.2. Data analysis

Data extraction and quality assessment were independently performed by two reviewers (LL, KD), and discrepancies were resolved by consultation with a third reviewer (SV). We extracted the data at meta-analysis and individual study levels (Supplementary Table S1). We used the revised AMSTAR (A Measurement Tool to Assess Systematic Reviews) 2 tool (18) to assess the methodological quality of included meta-analyses (high, moderate, low, and critically low).

To grade the strength of evidence for each association, we used pre-defined criteria from previous umbrella reviews (15–17, 19). Each association that presented statistically significant random-effects summary effect sizes (i.e., *p* ≤ 0.05) was graded as having convincing (class I), highly suggestive (class II), suggestive (class III), or weak (class IV) evidence based on the sample size, statistical significance, heterogeneity, small-study effect, and prediction interval (PrI). The classification criteria details are presented in Supplementary Table S4.

For each association, we extracted the effect sizes of individual studies included in each meta-analysis. For the meta-analysis which included individual studies in both the US and UK, we selected only meta-analyses that included individual studies conducted in the US. We recalculated the pooled effect sizes and 95% confidence intervals (CIs) using a random-effects model (20). Heterogeneity was assessed with the *I*^2^ statistic (21). We estimated the 95% PrI, which evaluated the uncertainty for the effect size that would be anticipated in a new study addressing the identical association (22). The evidence for small-study effects was assessed using Egger’s test (23). A value of *p* of less than 10 was taken as statistical evidence of the presence of small-study effects. For each association from meta-analyses initially graded as convincing or highly suggestive, we performed sensitivity analyses to determine the robustness of the findings, excluding small-size studies (<25th percentile) (24) and studies of low quality (25).

All statistical analyses were conducted using Stata software, version 17·0 (StataCorp LLC). The significance level was set at 2-sided *p* = 0.05 for all tests, except for the Egger’s tests, which had significant levels of 2-sided *p* = 0.10. The study protocol was registered in PROSPERO, CRD42022336805.

### 2.3. Role of the funding source

The funders of the study had no role in study design, data collection, data analysis, data interpretation, or writing of the report.

## 3. Results

**Figure 1:**
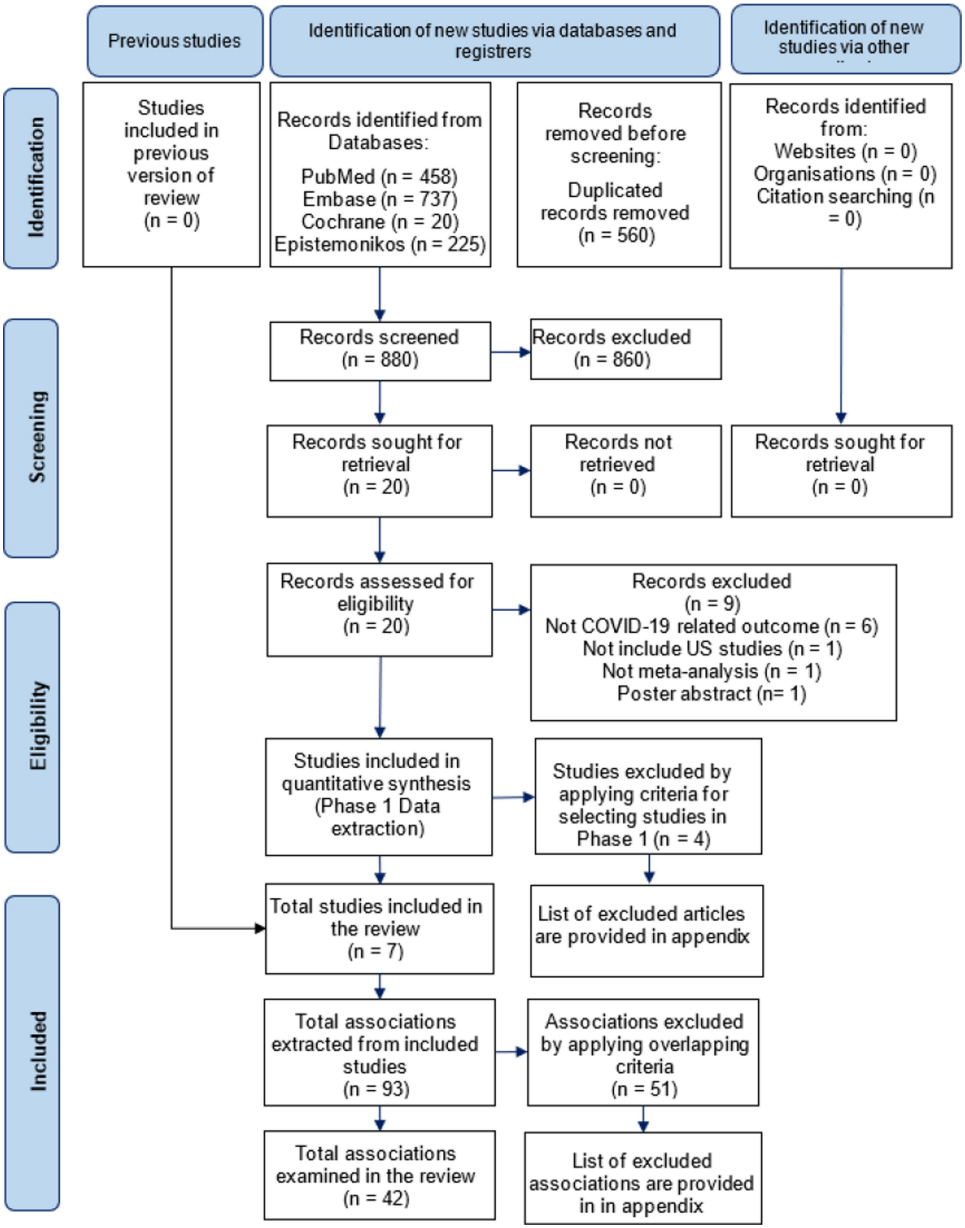
PRISMA flowchart of study selection process.

In total, we identified 1,440 publications, assessed full text 20 articles (1.4%), and included 7 meta-analyses eventually (0.5%). The reasons for excluding 1,433 articles (99.5%) are provided in Figure 1 and Supplementary Tables S2, S3. The flowchart of study selection is provided in Figure 1.

Our umbrella review included seven meta-analyses (4, 6–8, 26–28) published between November 2020 and February 2022. Those meta-analyses describe 42 associations among race and ethnicity and COVID-19 outcomes. The median number of studies contributing to each association was 4 (Interquartile range (IQR), 3–8). Among the 42 associations, three associations (7%) (7, 8) included COVID-19 infections among the general population and different race and ethnicity. Six (14%) (8, 28), five (12%) (4, 6–8), and three (7%) (8) associations included COVID-19 hospitalization, COVID-19 ICU admission, and severe COVID-19 among those infected, respectively. Sixteen (38%) (26) associations focused on COVID-19 readmission among those hospitalized. Among them, the outcome was readmissions more than 30 days in four associations (10%) (26), readmission <30 days in four (10%) (26) readmission to hospital or ICU in four (10%) (26), and hospital-only readmission in the remaining four associations (10%) (26). COVID-19 mortality among the infected and hospitalized patients was described in two (5%) (7) and three (7%) (8) associations, respectively. One (2%) (6) association focused on acute kidney injuries, and two (5%) (6, 7) focused on invasive mechanical ventilation among COVID-19 confirmed infection. Only one (2%) (27) association focused on COVID-19 severity among infected pregnant patients.

Regarding race and ethnicity, nine (21%) (6–8) associations compared COVID-19-related outcomes between White and Black individuals, seven (17%) (6–8) associations compared COVID-19-related outcomes between White and Hispanic individuals, six (14%) (4, 7, 8) associations compared COVID-19-related outcomes between White and Asian individuals, and six (14%) (26–28) associations compared COVID-19-related outcomes between White and non-White individuals. Five (12%) (26) associations compared COVID-19-related outcomes Hispanic and non-Hispanic individuals, while five (12%) (26, 28) COVID-19-related outcomes in Black and non-Black individuals, and four (10%) compared COVID-19-related outcomes between Asian and non-Asian (26) individuals. Additionally, all meta-analyses were to individual studies conducted in 2020. Additional descriptive characteristics and details of adjusted factors made for measurement and the time period studies conducted of each association in the meta-analysis are available in Supplementary Table S6. All associations examined in the review are provided in Supplementary Table S5.

Using the AMSTAR-2 tool, we classified one meta-analysis as high quality (27), one as moderate quality (6), and one as low quality (28). We classified the remaining four meta-analyses as critically low quality (4, 7, 8, 26) (Supplementary Table S5). Of the four critically low-quality meta-analyses, three (7, 8, 26) did not report evidence accounting for the risk of bias in individual studies when interpreting or discussing the results of the review, and one (4) did not provide a protocol.

**Figure 2:**
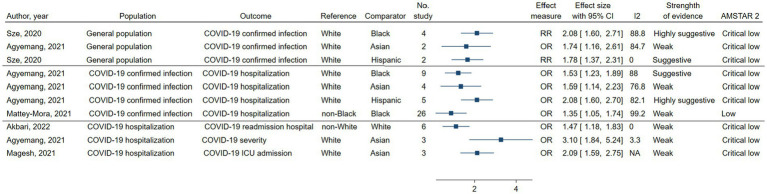
Summary of significant associations with class of evidence (highly suggestive, suggestive, weak) of race and ethnicity and COVID-19 related outcomes in the meta-analyses. ES, effect size; CI, confidence interval; RR, odds ratio; RR, risk ratio.

Ten of the 42 (24%) associations between race and ethnicity and COVID-19-related outcomes were statistically significant at *p* ≤ 0.05 (4, 7, 8, 26, 28). Of these, only two associations (5%) reached statistical significance at *p* ≤ 1.0 × 10^−6^ (Figure 2 and Supplementary Table S6) (7, 8). Twenty-five associations (60%) had high heterogeneity (*I*^2^ > 50%) (4, 6–8, 26, 28). The 95% PrI excluded the null value for only one association (2%) (26). Small-study effects were found in 8 (19%) associations (8, 26, 28). Summaries of all significant and non-significant associations are provided in Supplementary Table S5.

Of the ten significant associations, no association between race and ethnicity and COVID-19 outcomes was supported by convincing evidence. Two of the statistically significant associations (7, 8) (5%) were highly suggestive, two (7, 8) (5%) were suggestive, and six (4, 8, 26, 28) (14%) were weak (Table 1 and Figure 2). One association with highly suggestive evidence showed an increased risk of COVID-19 infection among the general population in Black compared to White individuals (RR, 2.08, 95%CI, 1.60–2.71) (7) (Figure 2, Supplementary Figure S1). This association remained suggestive with the conservative estimates from the sensitivity analysis where we excluded studies with small size (Supplementary Table S7). Another association between race and ethnicity and COVID-19 hospitalization among COVID-19 confirmed patients was supported by highly suggestive evidence, which revealed that individuals of Hispanic ethnicity were at a higher risk of hospitalization than their White counterparts (OR, 2.08, 95%CI, 1.60–2.70) (8) (Table 1 and Supplementary Figure S2). This association retained the same evidence ranking after sensitivity analyses (Supplementary Table S7).

**Table 1:** All significant associations of race and ethnicity and COVID-19 related outcomes in included meta-analyses.

Author, Year	Population	Outcome	Setting	Ref.	Comp.	No. of studies in US	Effect measure	ES (95%CI)	*p* value	*I*^2^ (%)	No. cases > 1,000	Largest study (95% CI)	Prediction Interval	Small study effect	CE	AMSTAR 2
**Highly suggestive association**																
Sze, 2020 (7)	General population	COVID-19 confirmed infection	US, UK	White	Black	4	RR	2.08 (1.6–2.71)	5.1 × 10^−8^	88.8	Yes	2.48 (1.68–3.66)	0.62–6.96	No	II	Critical low
Agyemang, 2021 (8)	COVID-19 confirmed infection	COVID-19 hospitalization	US	White	Hispanic	5	OR	2.08 (1.60–2.70)	3.7 × 10^−8^	82.1	Yes	2,13 (2.12–2.14)	0.87–4.97	No	II	Critical low
**Suggestive association**																
Sze, 2020 (7)	General population	COVID-19 confirmed infection	US, UK	White	Hispanic	2	RR	1.78 (1.37–2.31)	1.3 × 10^−5^	0.0	Yes	1.68 (1.18–2.33)	NA	NA	III	Critical low
Agyemang, 2021 (8)	COVID-19 confirmed infection	COVID-19 hospitalization	US	White	Black	9	OR	1.53 (1.23–1.89)	1.0 × 10^−4^	88	Yes	1.68 (1.63–1.73)	0.74–3.13	No	III	Critical low
**Weak association**																
Agyemang, 2021 (8)	General population	COVID-19 confirmed infection	US	White	Asian	2	OR	1.74 (1.16–2.61)	7.1 × 10^−3^	84.7	NA	2.16 (1.68–2.79)	NA	NA	IV	Critical low
Agyemang, 2021 (8)	COVID-19 confirmed infection	COVID-19 hospitalization	US	White	Asian	4	OR	1.59 (1.14–2.23)	6.6 × 10^−3^	76.8	NA	1.29 (0.97–1.72)	0.37–6.89	No	IV	Critical low
Mattey-Mora, 2021 (28)	COVID-19 confirmed infection	COVID-19 hospitalization	Mixed	non-Black	Black	26	OR	1.35 (1.05–1.74)	0.018	99.2	NA	0.97 (0.93–1)	0.38–4.83	No	IV	Low
Akbari, 2022 (26)	COVID-19 hospitalization	COVID-19 readmission hospital	US, UK	non-White	White	6	OR	1.47 (1.18–1.83)	0.001	0.0	No	0.88 (0.45–1.72)	1.07–2.01	Yes	IV	Critical low
Agyemang, 2021 (8)	COVID-19 hospitalization	COVID-19 severity	US	White	Asian	3	OR	3.11 (1.84–5.24)	2.2 × 10^−5^	3.3	No	2.13 (0.82–5.55)	0.08,114.51	No	IV	Critical low
Magesh, 2021 (4)	COVID-19 hospitalization	COVID-19 ICU admission	US	White	Asian	3	OR	2.09 (1.59–2.76)	NA	NA	NA	NA	NA	NA	IV	Critical low

OR, odds ratio; RR, risk ratio; NA, not available; NS, non-significant; Ref., reference; Comp., comparator; E.S., effect size; CI, confidence interval; C.E., class of evidence.

Two associations were graded suggestive. The first was the association between Hispanic and White individuals regarding the risk of COVID-19 infection (RR, 1.78, 95%CI, 1.37–2.31) (7). The second was the association between Black and White individuals in terms of the risk of COVID-19 hospitalization among those infected with COVID-19 (OR, 1.53, 95%CI, 1.23–1.89) (8) (Table 1). Individuals of Asian race were at a higher risk of COVID-19 infection (OR, 1.74, 95%CI, 1.16–2.61) (8), hospitalization (OR, 1.59, 95%CI, 1.14–2.23) (8), severe COVID-19 (OR, 3.11, 95%CI, 1.84–5.24) (8), and ICU admission (OR, 2.09, 95%CI, 1.59–2.76) (8) compared to White individuals. However, all these associations were classified as having weak evidence. One study found weak evidence for an increased risk of COVID-19 hospitalization among Black versus non-Black individuals (OR, 1.35, 95%CI, 1.05–1.74) (28). The association between COVID-19 readmission to hospital and race and ethnicity existed in one meta-analysis supported by a weak level of evidence (White versus non-White, OR, 1.47, 95%CI, 1.18–1.83) (26). More information on ten significant associations is provided in Figure 2 and Table 1.

Notably, among five associations examining disparities in mortality among those infected, no evidence of a difference was found between minority racial groups and White populations in the COVID-19 mortality risk among those infected (Black vs. White: OR, 1.03, 95%CI, 0.9–1.17) (7) and those hospitalized (Asian vs. White: OR, 0.96, 95%CI, 0.78–1.17) (8) (Supplementary Table S5).

## 4. Discussion

This umbrella review systematically summarizes evidence of disparities in COVID-19-related outcomes between underrepresented groups in the US (Black, Hispanic, and Asian) and their White counterparts, spanning the continuum of care from outpatient cases to hospitalized COVID-19 patients. We examined seven published meta-analyses, which generated forty-two estimates for the associations of race and ethnicity with COVID-19-related outcomes. These associations between race and ethnicity and COVID-19-related outcomes were categorized into three levels, including COVID-19 outcomes in the general population, COVID-19 outcomes conditional on having COVID-19 infection, and COVID-19 outcomes conditional on being hospitalized for COVID-19 infection.

**Figure 3:**
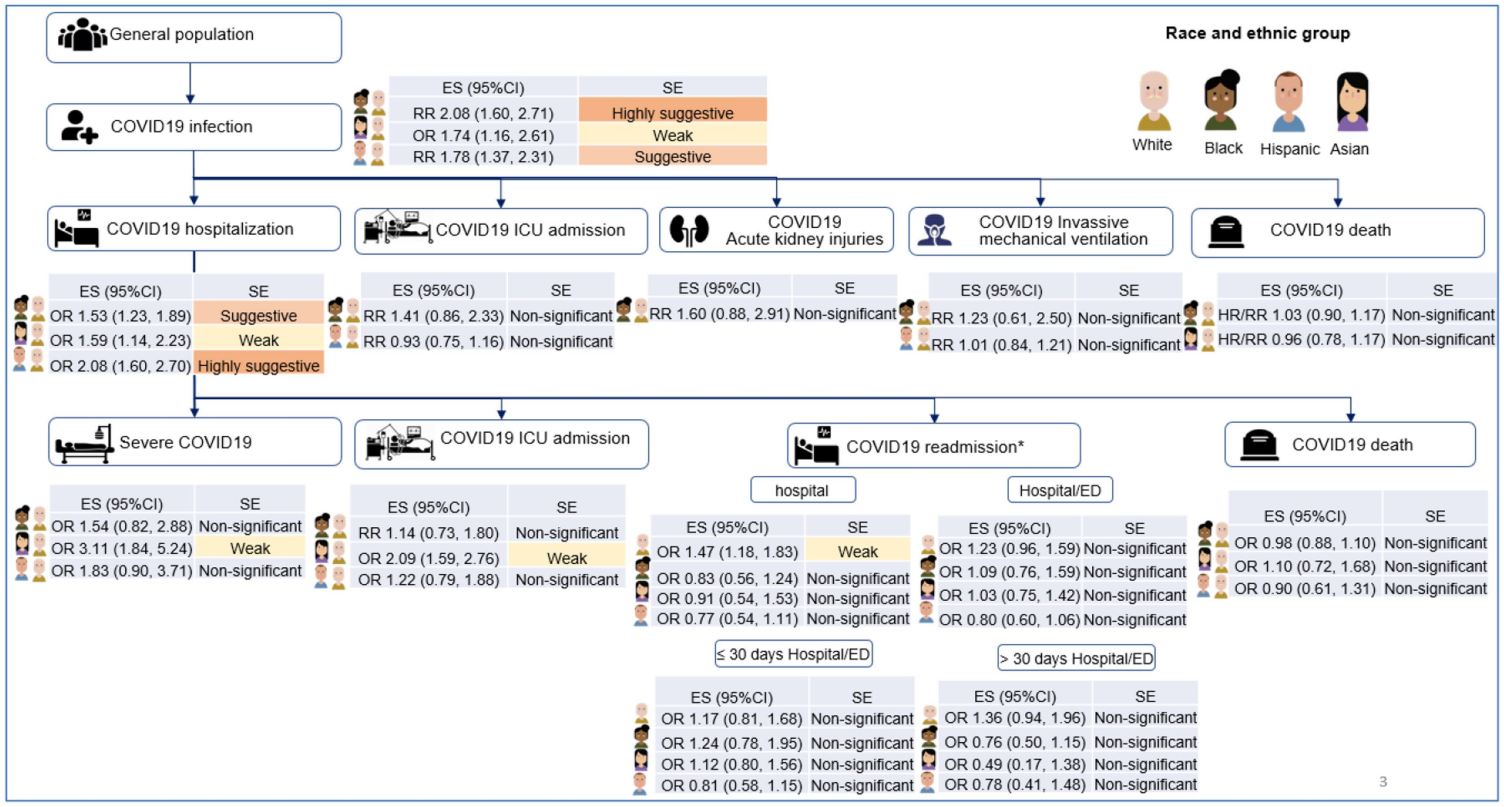
Illustration of the associations between race and ethnicity and main COVID-19 outcomes in included meta-analyses. ES, effect size; CI, confidence interval; RR, odds ratio; RR, risk ratio; HR, hazard ratios; SE, strength of evidence; NS, non-significant, *These associations compared White vs. non-White, Black vs. non-Black, Asian vs. non-Asian, and Hispanic vs. non-Hispanic.

Our findings show that strong evidence demonstrated that racial and ethnic minority groups had higher rates of COVID-19 infection, and higher risk of hospitalization once they were COVID-19 infected than White populations. However, no significant disparities were observed in COVID-19 mortality outcomes once patients were hospitalized. Three levels of COVID-19 outcomes in different populations are illustrated Figure 3.

Our results showed that Black and Hispanic individuals had a higher risk of COVID-19 infection compared to White individuals, consistent with previous studies (29–32). One possible reason for this disparity is that multi-generational living situations are more common among racial and ethnic minority groups due to living cost savings and/or cultures. These crowded environments can increase the risk of COVID-19 transmission (33–35). Another potential reason is that racial and ethnic minorities are more likely to live in overcrowded households or shared accommodations (29). Thus, isolation is simply more difficult from a practical standpoint among this population. A third possible reason for these disparities is that individuals from ethnic minority groups are more likely to work in occupations in essential industries in which they are in close proximity to others, thus, increasing their risk of exposure to COVID-19 (30, 36). Fourth, there may be language barriers that present challenges to the public health messaging regarding COVID-19 prevention (31, 37–39). Accessing facemasks and respiratory protective equipment fit was also a challenge for minorities, making the increasing risk for COVID-19 infections (34, 40).

When we examined COVID-19 outcomes conditional on being COVID-19 infected, we found that racial and ethnic groups also experienced a higher risk of hospitalization. One potential reason is that minorities have a higher burden of chronic health conditions, including diabetes, heart disease, and lung disease which can contribute to poor COVID-19 outcomes (32, 36, 41). Additionally, ethnic minority individuals are more likely to be uninsured (42), limiting their access to treatment services when infected (43). Consequently, they may suffer delays in obtaining testing and treatments until they are sicker and require hospitalization. Finally, implicit bias and structural racism, identified in multiple articles before and during the pandemic, might represent fundamental causes of disparities among different racial and ethnic groups (44, 45).

These factors relate to the conditions in which people are born, grow, live, work, and age, which are defined as social determinants of health (SDH) (34, 36, 46). These determinants influenced the unequal distribution of resources, money, and power, resulting in health disparities among groups of people, especially people with disadvantages (47). Some studies show that minority race/ethnicity accounted for a high proportion at low levels of some social determinant of health index (48, 49). The relationships between SDH, race and ethnicity, and COVID-19 outcomes are extremely complex (50) and have not been addressed in this umbrella review due to limited data reported in these included reviews. Future surveillance activities should incorporate race and ethnicity and SDH factors to examine this relationship.

Our umbrella review did not observe significant evidence of disparity in COVID-19 outcomes by racial and ethnic groups among those hospitalized. Notably, no significant associations regarding COVID-19 death among those who infected and those who got hospitalized were revealed in this review. The previous meta-analysis also showed similar patterns (25). Another study by Ioannou and colleagues investigated a large sample of 88,747 veterans who tested positive for COVID-19 and also found that Black and Hispanic patients did not have a statistically significant greater likelihood of death than White patients (41). However, some studies reported that ethnic minorities had a higher mortality rate than White individuals, which seems to contrast with our findings (51, 52). One possible explanation is that our review focused on mortality rates in patients with COVID-19 infection and those hospitalized for COVID-19 rather than in the general population. Thus, the increased mortality reported in those studies may reflect the increased of infection and hospitalization rather than an increased risk of death once hospitalized.

Our study confirms that strong evidence supports the existence of racial and ethnic disparities in COVID-19 infection rates, and hospitalization rates once infected (pre-hospitalization stage). Efforts to reduce poverty, protect essential workers, improve public health outreach to minority workers, and improve access to health care are needed to reduce the disparities in health outcomes. The lack of convincing evidence of disparities in outcomes after hospitalization is encouraging. Therefore, focusing on improving baseline existing disparity in our system may improve downstream health outcomes.

This is the first umbrella review comprehensively synthesizing and quantifying the evidence on the association between ethnicity and COVID-19 outcomes. Robust grading of the previous meta-analyses supports policymakers in considering race and ethnicity in future public health interventions, informing decisions regarding risk stratification at work, shielding advice, and informing strategies for allocating treatment and vaccinations. These findings may also be helpful for clinical practitioners in evaluating at-risk patients.

This umbrella review has several limitations. Firstly, due to limited data, some associations between COVID-19-related outcomes and some races and ethnicities were not included in these meta-analyses. Second, the quality of all primary studies included in each meta-analysis relied on the assessment reported by the respective meta-analysis. Additionally, our findings of weak or non-significant associations between race and ethnicity and severe COVID-19, ICU admission, or death among hospitalized patients could be due to the small number of studies investigating these associations. Future studies, hence, are needed to examine these associations. Lastly, the timeline of the meta-analyses and the data collection period of the studies included in each meta-analysis are also limitations of our review. These analyses were conducted across all pandemic stages in 2020 without classifying the specific waves of the pandemic. The associations between race and ethnicity and outcomes may vary depending on the waves of pandemics, in which, other different factors, such as COVID-19 variants and compliance with public health policies, may play roles in these relationships. Additionally, at that time, the COVID-19 vaccine was not available. Therefore, the associations reported here might change with the introduction of vaccines. Hence, these results should be interpreted with caution, and further studies which explore the outcome disparities after the COVID-19 vaccine era are needed.

The risks of COVID-19 infection among the general population and the risk of hospitalization among COVID-19 confirmed patients were higher in most ethnic minorities, but once hospitalized, no significant disparities were found in COVID-19 outcomes, especially mortality. These findings highlight racial and ethnic disparities in the pre-hospitalization stage and reinforce our perceptions about race and ethnicity inequity during the COVID-19 pandemic.

## Data availability statement

The original contributions presented in the study are included in the article/Supplementary material, further inquiries can be directed to the corresponding author.

## Author contributions

LL, KD, SV, PS, WW, RN, MF, BJ, AP, MJ, MS, and NC conceptualized the study. LL, KD, SV, and NC designed, planned, and oversaw the study. PS and WW did a pilot search. LL and KD performed comprehensively searched the literature, applied eligibility criteria, extracted data, conducted the risk of bias assessment and grade strength of evidence, and wrote the first draft of the manuscript. Disagreements were resolved with SV and NC. LL, KD, PS, and WW did the statistical analyses. LL, KD, SV, PS, WW, RN, MF, BJ, AP, MJ, MS, and NC contributed to the interpretation, reviewed, commented on the manuscript draft, and approved it for submission. All authors contributed to the article and approved the submitted version.

## Funding

Centers for Disease Control and Prevention (CDC).

## Conflict of interest

The authors declare that the research was conducted in the absence of any commercial or financial relationships that could be construed as a potential conflict of interest.

## Publisher’s note

All claims expressed in this article are solely those of the authors and do not necessarily represent those of their affiliated organizations, or those of the publisher, the editors and the reviewers. Any product that may be evaluated in this article, or claim that may be made by its manufacturer, is not guaranteed or endorsed by the publisher.
