# Supplementary material for: Disparities in COVID-19 related outcomes in the United States by race and ethnicity pre-vaccination era: an umbrella review of meta-analyses
Source: Front Public Health. 2023 Sep 7;11:1206988. doi: 10.3389/fpubh.2023.1206988 (PMC10513444; doi:10.3389/fpubh.2023.1206988)
Supplement: Supplementary file 2 [file Table_3.DOCX]

Supplementary Material

Disparities in COVID-19 Related Outcomes in the United States by Race and Ethnicity Pre-vaccination Era: An Umbrella Review of Meta-Analyses

**Lan M. Le, PhD ^1,+^, Khanh N.C. Duong, MSc ^1,+^, Sajesh K. Veettil, PhD ^1^, Pantakarn Saidoung, PharmD ^1^, Warintorn Wannaadisai, PharmD ^2^, Richard E. Nelson, PhD ^3,4^, Mike Friedrichs, MS ^5^, Barbara E. Jones, MD ^4,6^, Andrew T. Pavia, MD ^7^, Makoto M. Jones, MD ^3,4^, Prof. Matthew H. Samore, MD ^3,4^, Prof. Nathorn Chaiyakunapruk, PhD ^1,4,∗^**

^1^Department of Pharmacotherapy, College of Pharmacy, University of Utah, Salt Lake City, UT, USA

^2^Faculty of Pharmacy, Mahidol University, Bangkok, TL

^3^Division of Epidemiology, School of Medicine, University of Utah, Salt Lake City, UT, USA

^4^IDEAS Center, Veterans Affairs Salt Lake City Health Care System, Salt Lake City,

UT, USA

^5^Utah Department of Health, Salt Lake City, UT, USA

^6^Division of Pulmonary & Critical Care, University of Utah, Salt Lake City, UT, USA

^7^Division of Pediatric Infectious Diseases, University of Utah, Salt Lake City, Utah, USA

*** Correspondence:**Prof. Nathorn Chaiyakunapruk
[nathorn.chaiyakunapruk@utah.edu](mailto:nathorn.chaiyakunapruk@utah.edu)

# Supplementary Data: Search strategies

Databases:

1. PubMed

2. EMBASE

3. Cochrane Database of Systematic Reviews (CDSR)

4. Epistemonikos

## 1. PubMed (Search date: April 25, 2022)

| **No.** | **Search Strategies** |
| --- | --- |
| #1 | COVID-19 [MeSH Terms] |
| #2 | Coronavirus[MeSH Terms] |
| #3 | coronavirus*[tiab] |
| #4 | “corona virus*”[tiab] |
| #5 | covid19[tiab] |
| #6 | covid?19[tiab] |
| #7 | ncov?2019[tiab] |
| #8 | #1 OR #2 OR #3 OR #4 OR #5 OR #6 OR #7 |
| #9 | ethnic group[MeSH Terms] |
| #10 | ethnicit*[tiab] |
| #11 | ethnic[tiab] |
| #12 | race[tiab] |
| #13 | racial[tiab] |
| #14 | Hispanic[tiab] |
| #15 | African[tiab] |
| #16 | Asian[tiab] |
| #17 | black[tiab] |
| #18 | white[tiab] |
| #19 | Native American*[tiab] |
| #20 | Health Equity [MeSH Terms] |
| #21 | equit* [tiab] |
| #22 | inequit*[tiab] |
| #23 | equal*[tiab] |
| #24 | inequal*[tiab] |
| #25 | disparit*[tiab] |
| #26 | #9 OR #10 OR #11 OR #12 OR #13 OR #14 OR #15 OR #16 OR #17 OR #18 OR #19 OR #20 OR #21 OR #22 OR #23 OR #24 OR #25 |
| #27 | review, systematic[MeSH Terms] |
| #28 | “systematic review”[tiab] |
| #29 | “systematic literature review”[tiab] |
| #30 | “meta-analysis”[tiab] |
| #31 | “meta-analyses”[tiab] |
| #32 | “meta analysis”[tiab] |
| #33 | “meta analyses”[tiab] |
| #34 | #27 OR #28 OR #29 OR #30 OR #31 OR #32 OR #33 |
| #35 | #8 AND #26 AND #34 |

## EMBASE (Search date: April 25, 2022)

| **No.** | **Search Strategies** |
| --- | --- |
| #1 | 'covid 19'/exp |
| #2 | 'covid 19':ti,ab |
| #3 | covid19:ti,ab |
| #4 | covid?19:ti,ab |
| #5 | ncov?2019:ti,ab |
| #6 | coronavirus*:ti,ab |
| #7 | “corona virus*”:ti,ab |
| #8 | #1 OR #2 OR #3 OR #4 OR #5 OR #6 OR #7 |
| #9 | 'ethnic group'/exp |
| #10 | ethnicit*:ti,ab |
| #11 | ethnic:ti,ab |
| #12 | race: ti,ab |
| #13 | racial:ti,ab |
| #14 | hispanic:ti,ab |
| #15 | african:ti,ab |
| #16 | Asian:ti,ab |
| #17 | “Native American*”:ti,ab |
| #18 | black:ti,ab |
| #19 | white:ti,ab |
| #20 | ‘Health Equity’/exp |
| #21 | equit*:ti,ab |
| #22 | inequit*:ti,ab |
| #23 | equal*:ti,ab |
| #24 | inequal*:ti,ab |
| #25 | disparit*:ti,ab |
| #26 | #9 OR #10 OR #11 OR #12 OR #13 OR #14 OR #15 OR #16 OR #17 OR #18 OR #19 OR #20 OR #21 OR #22 OR #23 OR #24 OR #25 |
| #27 | 'systematic review'/exp |
| #28 | “systematic literature review”:ti,ab |
| #29 | “systematic review”:ti,ab |
| #30 | 'meta analysis'/exp |
| #31 | 'meta analysis':ti,ab |
| #32 | “meta-analysis”:ti,ab |
| #33 | “meta-analyses”:ti,ab |
| #34 | “meta analyses”:ti,ab |
| #35 | #27 OR #28 OR #29 OR #30 OR #31 OR #32 OR #33 OR #34 |
| #36 | #8 AND #26 AND #35 |

## Cochrane Database of Systematic Reviews (CDSR) (Search date: April 25, 2022)

| **No.** | **Search Strategies** |
| --- | --- |
| #1 | MeSH desciptor: [COVID-19] explore all trees |
| #2 | covid19:ti,ab,kw |
| #3 | covid?19:ti,ab,kw |
| #4 | ncov?2019:ti,ab,kw |
| #5 | coronavirus*:ti,ab,kw |
| #6 | “corona virus*”:ti,ab,kw |
| #7 | #1 OR #2 OR #3 OR #4 OR #5 OR #6 |
| #8 | ethnicit*: ti,ab,kw |
| #9 | ethnic:ti,ab,kw |
| #10 | race:ti,ab,kw |
| #11 | racial:ti,ab,kw |
| #12 | hispanic:ti,ab,kw |
| #13 | african:ti,ab,kw |
| #14 | Asian:ti,ab,kw |
| #15 | “Native American*”:ti,ab,kw |
| #16 | black:ti,ab,kw |
| #17 | white:ti,ab,kw |
| #18 | equit*:ti,ab,kw |
| #19 | inequit*:ti,ab,kw |
| #20 | equal*:ti,ab,kw |
| #21 | inequal*:ti,ab,kw |
| #22 | disparit*:ti,ab,kw |
| #23 | #8 OR #9 OR #10 OR #11 OR #12 OR #13 OR #14 OR #15 OR #16 OR #17 OR #18 OR #19 OR #20 OR #21 OR #22 |
| #24 | MeSH desciptor: [Systematic Reviews as Topic] explore all trees |
| #25 | MeSH desciptor: [Meta-Analysis as Topic] explore all trees |
| #26 | “systematic review” :ti,ab,kw |
| #27 | “systematic literature review” :ti,ab,kw |
| #28 | “meta-analysis” :ti,ab,kw |
| #29 | “meta-analyses” :ti,ab,kw |
| #30 | “meta analysis” :ti,ab,kw |
| #31 | “meta analyses” :ti,ab,kw |
| #32 | #24 OR #25 OR #26 OR #27 OR #28 OR #29 OR #30 OR #31 |
| #33 | #7 AND #23 AND #32 |

## Epistemonikos (Search date: April 25, 2022)

Title, abstract ((COVID?19 OR coronavirus* OR “corona virus*” OR covid19 OR ncov?2019) AND (ethnicit* OR ethnic OR race OR racial OR hispanic OR african OR Asian OR “Native American*” OR black OR white OR equit* OR inequit* OR equal* OR inequal* OR disparit*) AND (“systematic review” OR “systematic literature review” OR “meta-analysis” OR “meta-analyses” OR “meta analysis” OR “meta analyses”))

# Supplementary Figures

## Supplementary Figures 1

|  |
| --- |

**Supplementary Figure 1:** Forest plot of significant associations between COVID-19 confirmed infection and race and ethnicity.

Predictive interval for Black vs. White: 0·62–6·96, for Asian vs. White and Hispanic vs. White: no predictive interval is generated due to <3 studies. Note: CI: Confidence Interval

## Supplementary figure 2

|  |
| --- |

**Supplementary Figure 2:** Forest plot of significant associations between COVID-19 hospitalization and race and ethnicity

Predictive interval for Black vs. White: 0·74–3·13, for Asian vs. White: 0·37–6·89 and Hispanic vs. White: 0·87–4·97. Note: CI: Confidence Interval

## Supplementary figure 3

|  |
| --- |

**Supplementary Figure 3:** Forest plot of significant associations between COVID-19 hospitalization and race and ethnicity (Black vs. non-Black)

Predictive interval for Black vs. non-Black: 0·38–4·83. Note: ES: Effect size, CI: Confidence Interval

## Supplementary figure 4

|  |
| --- |

**Supplementary Figure 4:** Forest plot of significant associations between COVID-19 hospitalization and race and ethnicity (White vs. non-White)

Predictive interval for White vs. non-White: 1·07–2·01. Note: ES: Effect size, CI: Confidence Interval

## Supplementary figure 5

|  |
| --- |

**Supplementary Figure 5:** Forest plot of significant associations between severe COVID-19 and race and ethnicity (Asian vs. White)

Predictive interval for Asian vs. White: 0·08–114·51. Note: ES: Effect size, CI: Confidence Interval

## Supplementary figure 6

|  |
| --- |

**Supplementary Figure 6:** Forest plot of associations between COVID 19 infections and race and ethnicity (Black vs. White)

Predictive interval for primary analysis: 0·62–6·96, for sensitivity analysis: 0·07–52·37. Note: ES: Effect size, CI: Confidence Interval

## Supplementary figure 7

|  |
| --- |

**Supplementary Figure 7:** Forest plot of associations between COVID 19 hospitalization and race and ethnicity (Black vs. White)

Predictive interval for primary analysis: 0·74–3·13, for sensitivity analysis: 0·62–3·03. Note: ES: Effect size, CI: Confidence Interval.

## Supplementary figure 8

|  |
| --- |

**Supplementary Figure 8:** Forest plot of associations between COVID 19 hospitalization and race and ethnicity (Hispanic vs. White)

Predictive interval for primary analysis: 0·87–4·97, for sensitivity analysis: 0·59–7·52. Note: ES: Effect size, CI: Confidence Interval.

# Supplementary Tables

## Supplementary Table 1: Selection and Data Extraction Process

| **1. Selection Process** | |
| --- | --- |
| **Review Question** | Are COVID-19 related outcome disparities in the United States associated with race and ethnicity?  How reliable is the evidence behind these associations in published meta-analyses? |
| **Objective** | To summarize all evidence based on existing meta-analyses to answer questions about the direction, magnitude, significance, and certainty of the evidence of the associations between race and ethnicity and various COVID-19-related outcomes in the US setting. |
| **Inclusion criteria** | **PICO** Participant: The population was defined according to the original meta-analysis studies.  Exposure: Racial/Ethnic minority groups. This was guided by how studies categories and define ethnic minority groups  Comparator: Majority racial/ethnic groups, depending on the study/country. The race/ethnic group were classified according to original meta-analyses, which mostly classified as White, Asian, Black, and Hispanic group (Hispanic people may be of any race but are categorized as Hispanic; other groups are non-Hispanic)  Outcome: Any COVID-19-related outcomes associated with race and ethnicity as reported in the original meta-analysis studies.  The article was selected based on the following criteria:  1) The study is a meta-analysis study  2) The study reported COVID-19 related outcomes for different race/ ethnicity  3) The meta-analysis includes studies conducted on the US population |
| **Exclusion criteria** | We excluded meta-analyses that provided insufficient or inadequate data for quantitative synthesis. Meta-analyses that reported COVID-19-related outcomes in populations other than the US population were also excluded. |
| **Search** | We used the search strategies which combined the search terms related to COVID-19, race and ethnicity, health inequity/disparity, and systematic review/meta-analysis. The reference lists of selected studies were also explored to identify further relevant studies which were unable to be identified through electronic searching. There were no restrictions on time and language in searching |
| **2. Data extraction** | |
| **Preliminary data extraction (phase I)** | - For each eligible meta-analysis, we extracted the following data independently: first author, year of publication, study setting, study design, specific population under investigation, specific COVID-19 related outcomes, numbers of studies in meta-analysis, numbers of studies in the US, ethnicity reference group, ethnicity comparator groups, number of adjusted factors (if reported), lists of adjusted factors (if reported)   **Selection between overlapping meta-analyses:**   - When more than one meta-analysis on the same association was available, the meta-analysis with the largest dataset and reported adjusted effect-size with a comprehensive set of confounding variables was selected, particularly, the meta-analysis with the largest number of primary studies were selected. If more than one published meta-analysis on the same association included an equal number of studies, the one with the largest number of cases were chosen. If more than one published meta-analysis fulfilled both criteria, the one with more available information on primary studies were selected [[1-4](#_ENREF_1)]. |
| **Data extraction for evidence synthesis (Phase II data synthesis)** | We extracted the following data at both the meta-analysis and individual study levels   - Meta-analysis level: First author, year of publication, study setting, study design, specific population under investigation, specific COVID-19 related outcomes, numbers of studies in meta-analysis, ethnicity reference group, ethnicity comparator groups, total number of cases and participants in each group, the stated summary meta-analytic estimates and their corresponding 95% confidence interval (95% CI), number of adjusted factors (if reported), lists of adjusted factors (if reported), evidence grading (if reported), and quality score according to AMSTAR 2. - Individual study level: first author, year of publication, study setting, study design, specific population under investigation, specific COVID-19 related outcomes, ethnicity reference group, ethnicity comparator groups, the total number of cases and participants in each group, the stated summary meta-analytic estimates and their corresponding 95% confidence interval (95% CI), quality assessment (if reported).   Microsoft Excel spreadsheet (XP professional edition; Microsoft Corp) will be used in data extraction |
| **Quality assessment** | To assess the methodological quality of each meta-analysis (high, moderate, low, and critically low), we used the revised AMSTAR 2 tool, a 16-item instrument to assess the methodological quality of systematic reviews of randomized and nonrandomized studie s[[5](#_ENREF_5)].To grade the credibility of evidence per association, we used the pre-defined criteria which were used in previous umbrella reviews. The credibility of each association was graded as having convincing (class 1), highly suggestive (class 2), suggestive (class 3), or weak (class 4) evidence according to the information on the threshold of p-value in statistical tests, number of cases, small-study effect, and prediction interval [[2-4](#_ENREF_2), [6](#_ENREF_6)]. The risk of bias in primary cohort studies was reported as it was originally stated by the author. |

## Supplementary Table 2: Excluded meta-analysis

| **No.** | **Author** | **Year of publication** | **Title** | **Reason for exclusion** |
| --- | --- | --- | --- | --- |
| 1 | Alhumaid [[7](#_ENREF_7)] | 2021 | Diabetic ketoacidosis in patients with SARS-CoV-2: a systematic review and meta-analysis | Unable to identify US studies per association |
| 2 | Bhakta [[8](#_ENREF_8)] | 2022 | A systematic review and meta-analysis of racial disparities in deep vein thrombosis and pulmonary embolism events in patients hospitalized with coronavirus disease 2019 | Did not report OR/RR/HR |
| 3 | Khan [[9](#_ENREF_9)] | 2021 | Differences in pregnancy and perinatal outcomes among symptomatic versus asymptomatic COVID-19-infected pregnant women: a systematic review and meta-analysis | Fewerthan two US studies per association |
| 4 | Mude [[10](#_ENREF_10)] | 2021 | Racial disparities in COVID-19 pandemic cases, hospitalisations, and deaths: A systematic review and meta-analysis | Did not report OR/RR/HR |

## Supplementary Table 3: Selection of unique associations for overlapping meta-analyses

| **Author, Year** | **Population** | **Outcome** | **Setting** | **Reference** | **Comparator** | **No. of studies** | **No. of studies in US** | **Effect measure** | **No. of adjusted factors** | **Adjusted factors** | **Association**  **ID** | **Select association** | **Reason for being selected** |
| --- | --- | --- | --- | --- | --- | --- | --- | --- | --- | --- | --- | --- | --- |
| Agyemang, 2021[[11](#_ENREF_11)] | General population | COVID-19 confirmed infection | US | White | Black | 3 | 3 | adjusted OR | ≥ 3 | at least 3 factors are adjusted: Age, sex, comorbidities | 1 |  |  |
| Magesh, 2021[[12](#_ENREF_12)] | General population | COVID-19 confirmed infection | US | White | Black | 20 | 20 | adjusted OR | 2 | age, sex | 1 |  |  |
| Magesh, 2021[[12](#_ENREF_12)] | General population | COVID-19 confirmed infection | US | White | Black | 20 | 20 | adjusted OR | 1 | sex | 1 |  |  |
| Magesh, 2021[[12](#_ENREF_12)] | General population | COVID-19 confirmed infection | US | White | Black | 20 | 20 | adjusted OR | 1 | Area deprivation index (ADI) | 1 |  |  |
| Magesh, 2021[[12](#_ENREF_12)] | General population | COVID-19 confirmed infection | US | White | Black | 20 | 20 | adjusted OR | 1 | income | 1 |  |  |
| Magesh, 2021[[12](#_ENREF_12)] | General population | COVID-19 confirmed infection | US | White | Black | 20 | 20 | adjusted OR | 1 | clinical care | 1 |  |  |
| Magesh, 2021[[12](#_ENREF_12)] | General population | COVID-19 confirmed infection | US | White | Black | 20 | 20 | adjusted OR | 1 | Comorbidities | 1 |  |  |
| Sze, 2020[[13](#_ENREF_13)] | General population | COVID-19 confirmed infection | US, UK | White | Black | 8 | 4 | adjusted RR | ≥2 | at least 2 factors: age, sex. A prior set of confounders: age, sex, deprivation, obesity, and comorbidities | 1 | select | largest number of studies + information of individual studies |
| Agyemang, 2021[[11](#_ENREF_11)] | General population | COVID-19 confirmed infection | US | White | Asian | 2 | 2 | adjusted OR | ≥ 3 | at least 3 factors are adjusted: Age, sex, comorbidities | 2 | select | largest number of studies + largest number of adjusted factors + information of individual studies |
| Magesh, 2021[[12](#_ENREF_12)] | General population | COVID-19 confirmed infection | US | White | Asian | NA | NA | adjusted OR | 1 | sex | 2 |  |  |
| Magesh, 2021[[12](#_ENREF_12)] | General population | COVID-19 confirmed infection | US | White | Asian | 7 | 7 | adjusted OR | 1 | Area deprivation index (ADI) | 2 |  |  |
| Magesh, 2021[[12](#_ENREF_12)] | General population | COVID-19 confirmed infection | US | White | Asian | 7 | 7 | adjusted OR | 1 | income | 2 |  |  |
| Magesh, 2021[[12](#_ENREF_12)] | General population | COVID-19 confirmed infection | US | White | Asian | 7 | 7 | adjusted OR | 1 | clinical care | 2 |  |  |
| Magesh, 2021[[12](#_ENREF_12)] | General population | COVID-19 confirmed infection | US | White | Asian | 7 | 7 | adjusted OR | 1 | Urban Core Opportunity Index (UOI) | 2 |  |  |
| Sze, 2020[[13](#_ENREF_13)] | General population | COVID-19 confirmed infection | US, UK | White | Asian | 5 | 2 | adjusted RR | ≥2 | at least 2 factors: age, sex. A prior set of confounders: age, sex, deprivation, obesity, and comorbidities | 2 |  |  |
| Magesh, 2021[[12](#_ENREF_12)] | General population | COVID-19 confirmed infection | US | White | Hispanic | NA | NA | adjusted OR | 2 | age, sex | 3 |  |  |
| Magesh, 2021[[12](#_ENREF_12)] | General population | COVID-19 confirmed infection | US | White | Hispanic | NA | NA | adjusted OR | 1 | sex | 3 |  |  |
| Magesh, 2021[[12](#_ENREF_12)] | General population | COVID-19 confirmed infection | US | White | Hispanic | 10 | 10 | adjusted OR | 1 | Area deprivation index (ADI) | 3 |  |  |
| Magesh, 2021[[12](#_ENREF_12)] | General population | COVID-19 confirmed infection | US | White | Hispanic | 10 | 10 | adjusted OR | 1 | income | 3 |  |  |
| Magesh, 2021[[12](#_ENREF_12)] | General population | COVID-19 confirmed infection | US | White | Hispanic | 10 | 10 | adjusted OR | 1 | Urban Core Opportunity Index (UOI) | 3 |  |  |
| Magesh, 2021[[12](#_ENREF_12)] | General population | COVID-19 confirmed infection | US | White | Hispanic | 10 | 10 | adjusted OR | 1 | Comorbidities | 3 |  |  |
| Sze, 2020[[13](#_ENREF_13)] | General population | COVID-19 confirmed infection | US, UK | White | Hispanic | 3 | 2 | adjusted RR | ≥2 | at least 2 factors: age, sex. A prior set of confounders: age, sex, deprivation, obesity, and comorbidities | 3 | select | largest number of studies + information of individual studies |
| Agyemang, 2021[[11](#_ENREF_11)] | COVID-19 confirmed infection | COVID-19 hospitalization | US | White | Black | 9 | 9 | adjusted OR | ≥ 3 | at least 3 factors are adjusted: Age, sex, comorbidities | 4 | select | largest number of studies |
| Magesh, 2021[[12](#_ENREF_12)] | COVID-19 confirmed infection | COVID-19 hospitalization | US | White | Black | 4 | 4 | adjusted OR | 1 | ADI | 4 |  |  |
| Raharja, 2021[[14](#_ENREF_14)] | COVID-19 confirmed infection | COVID-19 hospitalization | US,UK | White | Black | 4 | 3 | adjusted RR | 4 | age, sex, comorbidities, SES | 4 |  |  |
| Agyemang, 2021[[11](#_ENREF_11)] | COVID-19 confirmed infection | COVID-19 hospitalization | US | White | Asian | 4 | 4 | adjusted OR | ≥ 3 | at least 3 factors are adjusted: Age, sex, comorbidities | 5 | select | largest number of studies |
| Raharja, 2021[[14](#_ENREF_14)] | COVID-19 confirmed infection | COVID-19 hospitalization | US,UK | White | Asian | 3 | 3 | adjusted RR | 3 | age, sex, comorbidities | 5 |  |  |
| Agyemang, 2021[[11](#_ENREF_11)] | COVID-19 confirmed infection | COVID-19 hospitalization | US | White | Hispanic | 5 | 5 | adjusted OR | ≥ 3 | at least 3 factors are adjusted: Age, sex, comorbidities | 6 | select | largest number of studies |
| Raharja, 2021[[14](#_ENREF_14)] | COVID-19 confirmed infection | COVID-19 hospitalization | US,UK | White | Hispanic | 3 | 3 | adjusted RR | 3 | age, sex, comorbidities | 6 |  |  |
| Mattey-Mora, 2021[[15](#_ENREF_15)] | COVID-19 confirmed infection | COVID-19 hospitalization | Worldwide | non-White | White | 27 | 25 | unadjusted OR | 0 |  | 7 | select | unique association |
| Mattey-Mora, 2021[[15](#_ENREF_15)] | COVID-19 confirmed infection | COVID-19 hospitalization | Worldwide | non-Black | Black | 27 | 26 | unadjusted OR | 0 |  | 8 | select | unique association |
| Mattey-Mora, 2021[[15](#_ENREF_15)] | COVID-19 confirmed infection | COVID-19 hospitalization | Worldwide | non-Hispanic | Hispanic | 22 | 22 | unadjusted OR | 0 |  | 9 | select | unique association |
| Raharja, 2021[[14](#_ENREF_14)] | COVID-19 confirmed infection | COVID-19 ICU admission | US, UK | White | Black | 3 | 3 | adjusted RR | 3 | age, sex, comorbidities | 10 | select | unique association |
| Raharja, 2021[[14](#_ENREF_14)] | COVID-19 confirmed infection | COVID-19 ICU admission | US, UK | White | Hispanic | 2 | 2 | adjusted RR | 3 | age, sex, comorbidities | 11 | select | unique association |
| Akbari, 2022[[16](#_ENREF_16)] | COVID-19 hospitalization | COVID-19 readmission ≤ 30 days (hospital/ED) | US, UK | non-White | White | 8 | 7 | unadjusted OR | 0 |  | 12 | select | unique association |
| Akbari, 2022[[16](#_ENREF_16)] | COVID-19 hospitalization | COVID-19 readmission ≤ 30 days (hospital/ED) | US, UK | non-Black | Black | 9 | 7 | unadjusted OR | 0 |  | 13 | select | unique association |
| Akbari, 2022[[16](#_ENREF_16)] | COVID-19 hospitalization | COVID-19 readmission ≤ 30 days (hospital/ED) | US, UK | Asian | non-Asian | 6 | 5 | unadjusted OR | 0 |  | 14 | select | unique association |
| Akbari, 2022[[16](#_ENREF_16)] | COVID-19 hospitalization | COVID-19 readmission ≤ 30 days (hospital/ED) | US, UK | non-Hispanic | Hispanic | 10 | 8 | unadjusted OR | 0 |  | 15 | select | unique association |
| Akbari, 2022[[16](#_ENREF_16)] | COVID-19 hospitalization | COVID-19 readmission > 30 days (hospital/ED) | US, UK | non-White | White | 3 | 3 | unadjusted OR | 0 |  | 16 | select | unique association |
| Akbari, 2022[[16](#_ENREF_16)] | COVID-19 hospitalization | COVID-19 readmission > 30 days (hospital/ED) | US, UK | non-Black | Black | 3 | 3 | unadjusted OR | 0 |  | 17 | select | unique association |
| Akbari, 2022[[16](#_ENREF_16)] | COVID-19 hospitalization | COVID-19 readmission > 30 days (hospital/ED) | US, UK | Asian | non-Asian | 3 | 3 | unadjusted OR | 0 |  | 18 | select | unique association |
| Akbari, 2022[[16](#_ENREF_16)] | COVID-19 hospitalization | COVID-19 readmission > 30 days (hospital/ED) | US, UK | non-Hispanic | Hispanic | 2 | 2 | unadjusted OR | 0 |  | 19 | select | unique association |
| Akbari, 2022[[16](#_ENREF_16)] | COVID-19 hospitalization | COVID-19 readmission hospital | US, UK | non-White | White | 9 | 6 | unadjusted OR | 0 |  | 20 | select | unique association |
| Akbari, 2022[[16](#_ENREF_16)] | COVID-19 hospitalization | COVID-19 readmission hospital | US, UK | non-Black | Black | 10 | 6 | unadjusted OR | 0 |  | 21 | select | unique association |
| Akbari, 2022[[16](#_ENREF_16)] | COVID-19 hospitalization | COVID-19 readmission hospital | US, UK | Asian | non-Asian | 8 | 5 | unadjusted OR | 0 |  | 22 | select | unique association |
| Akbari, 2022[[16](#_ENREF_16)] | COVID-19 hospitalization | COVID-19 readmission hospital | US, UK | non-Hispanic | Hispanic | 10 | 7 | unadjusted OR | 0 |  | 23 | select | unique association |
| Akbari, 2022[[16](#_ENREF_16)] | COVID-19 hospitalization | COVID-19 readmission hospital/ED | US, UK | non-White | White | 10 | 10 | unadjusted OR | 0 |  | 24 | select | unique association |
| Akbari, 2022[[16](#_ENREF_16)] | COVID-19 hospitalization | COVID-19 readmission hospital/ED | US, UK | non-Black | Black | 11 | 10 | unadjusted OR | 0 |  | 25 | select | unique association |
| Akbari, 2022[[16](#_ENREF_16)] | COVID-19 hospitalization | COVID-19 readmission hospital/ED | US, UK | Asian | non-Asian | 9 | 8 | unadjusted OR | 0 |  | 26 | select | unique association |
| Akbari, 2022[[16](#_ENREF_16)] | COVID-19 hospitalization | COVID-19 readmission hospital/ED | US, UK | non-Hispanic | Hispanic | 11 | 10 | unadjusted OR | 0 |  | 27 | select | unique association |
| Raharja, 2021[[14](#_ENREF_14)] | COVID-19 confirmed infection | acute kidney injuries | US, UK | White | Black | 2 | 2 | adjusted RR | 3 | age, sex, comorbidities | 28 | select | unique association |
| Raharja, 2021[[14](#_ENREF_14)] | COVID-19 confirmed infection | invasive mechanical ventilation | US, UK | White | Black | 3 | 3 | adjusted RR | 3 | age, sex, comorbidities | 29 | select | unique association |
| Raharja, 2021[[14](#_ENREF_14)] | COVID-19 confirmed infection | invasive mechanical ventilation | US, UK | White | Hispanic | 2 | 2 | adjusted RR | 3 | age, sex, comorbidities | 30 | select | unique association |
| Magesh, 2021[[12](#_ENREF_12)] | COVID-19 confirmed infection | COVID-19 death | US | White | Black | NA | NA | adjusted OR | 1 | sex | 31 |  |  |
| Magesh, 2021[[12](#_ENREF_12)] | COVID-19 confirmed infection | COVID-19 death | US | White | Black | 6 | 6 | adjusted OR | 1 | age | 31 |  |  |
| Magesh, 2021[[12](#_ENREF_12)] | COVID-19 confirmed infection | COVID-19 death | US | White | Black | 6 | 6 | adjusted OR | 1 | Area deprivation index (ADI) | 31 |  |  |
| Magesh, 2021[[12](#_ENREF_12)] | COVID-19 confirmed infection | COVID-19 death | US | White | Black | 6 | 6 | adjusted OR | 1 | income | 31 |  |  |
| Magesh, 2021[[12](#_ENREF_12)] | COVID-19 confirmed infection | COVID-19 death | US | White | Black | 6 | 6 | adjusted OR | 1 | clinical care | 31 |  |  |
| Magesh, 2021[[12](#_ENREF_12)] | COVID-19 confirmed infection | COVID-19 death | US | White | Black | 6 | 6 | adjusted OR | 1 | Comorbidities | 31 |  |  |
| Sze, 2020[[13](#_ENREF_13)] | COVID-19 confirmed infection | COVID-19 death | US, UK | White | Black | 18 | 15 | adjusted HR/RR | ≥2 | at least 2 factors: age, sex. A prior set of confounders: age, sex, deprivation, obesity, and comorbidities | 31 | select | largest number of studies |
| Raharja, 2021[[14](#_ENREF_14)] | COVID-19 confirmed infection | COVID-19 death | US, UK | White | Black | 6 | 5 | adjusted RR | 3 | age, sex, comorbidities | 31 |  |  |
| Magesh, 2021[[12](#_ENREF_12)] | COVID-19 confirmed infection | COVID-19 death | US | White | Asian | 4 | 4 | adjusted OR | 1 | Area deprivation index (ADI) | 32 |  |  |
| Magesh, 2021[[12](#_ENREF_12)] | COVID-19 confirmed infection | COVID-19 death | US | White | Asian | 4 | 4 | adjusted OR | 1 | income | 32 |  |  |
| Magesh, 2021 | COVID-19 confirmed infection | COVID-19 death | US | White | Asian | 4 | 4 | adjusted OR | 1 | clinical care | 32 |  |  |
| Sze, 2020[[13](#_ENREF_13)] | COVID-19 confirmed infection | COVID-19 death | US, UK | White | Asian | 6 | 3 | adjusted HR/RR | ≥2 | at least 2 factors: age, sex. A prior set of confounders: age, sex, deprivation, obesity, and comorbidities | 32 | select | largest number of studies + information of individual studies |
| Raharja, 2021[[14](#_ENREF_14)] | COVID-19 confirmed infection | COVID-19 death | US,UK | White | Asian | 3 | 2 | adjusted logHR | 3 | age, sex, comorbidities | 32 |  |  |
| Agyemang, 2021 [[11](#_ENREF_11)] | COVID-19 hospitalization | COVID-19 severity | US | White | Black | 3 | 3 | adjusted OR | ≥ 3 | at least 3 factors are adjusted: Age, sex, comorbidities | 33 | select |  |
| Agyemang, 2021 [[11](#_ENREF_11)] | COVID-19 hospitalization | COVID-19 severity | US | White | Hispanic | 2 | 2 | adjusted OR | ≥ 3 | at least 3 factors are adjusted: Age, sex, comorbidities | 34 | select | unique association |
| Agyemang, 2021 [[11](#_ENREF_11)] | COVID-19 hospitalization | COVID-19 severity | US | White | Asian | 3 | 3 | unadjusted OR | 0 | 0 | 35 | select |  |
| Agyemang, 2021 [[11](#_ENREF_11)] | COVID-19 hospitalization | COVID-19 ICU admission | US | White | Black | 4 | 4 | adjusted OR | ≥ 3 | at least 3 factors are adjusted: Age, sex, comorbidities | 36 |  |  |
| Magesh, 2021[[12](#_ENREF_12)] | COVID-19 hospitalization | COVID-19 ICU admission | US | White | Black | NA | NA | adjusted OR | 1 | sex | 36 |  |  |
| Magesh, 2021[[12](#_ENREF_12)] | COVID-19 hospitalization | COVID-19 ICU admission | US | White | Black | 4 | 4 | adjusted OR | 1 | income | 36 |  |  |
| Magesh, 2021[[12](#_ENREF_12)] | COVID-19 hospitalization | COVID-19 ICU admission | US | White | Black | 4 | 4 | adjusted OR | 1 | Urban Core Opportunity Index (UOI) | 36 |  |  |
| Magesh, 2021[[12](#_ENREF_12)] | COVID-19 hospitalization | COVID-19 ICU admission | US | White | Black | NA | NA | adjusted OR | 1 | sex | 36 |  |  |
| Sze, 2020[[13](#_ENREF_13)] | COVID-19 hospitalization | COVID-19 ICU admission | US, UK | White | Black | 4 | 3 | adjusted RR | ≥2 | at least 2 factors: age, sex. A prior set of confounders: age, sex, deprivation, obesity, and comorbidities | 36 | select | largest number of studies + information of individual studies |
| Magesh, 2021[[12](#_ENREF_12)] | COVID-19 hospitalization | COVID-19 ICU admission | US | White | Asian | NA | NA | adjusted OR | 1 | sex | 37 |  |  |
| Magesh, 2021[[12](#_ENREF_12)] | COVID-19 hospitalization | COVID-19 ICU admission | US | White | Asian | 3 | 3 | adjusted OR | 1 | age | 37 | select | most common adjusted factor |
| Magesh, 2021 | COVID-19 hospitalization | COVID-19 ICU admission | US | White | Asian | 3 | 3 | adjusted OR | 1 | income | 37 |  |  |
| Agyemang, 2021[[11](#_ENREF_11)] | COVID-19 hospitalization | COVID-19 ICU admission | US | White | Hispanic | 3 | 3 | adjusted OR | ≥ 3 | at least 3 factors are adjusted: Age, sex, comorbidities | 38 | select | largest number of studies + information of individual studies + largest number of adjusted factors |
| Magesh, 2021[[12](#_ENREF_12)] | COVID-19 hospitalization | COVID-19 ICU admission | US | White | Hispanic | NA | NA | adjusted OR | 1 | sex | 38 |  |  |
| Magesh, 2021[[12](#_ENREF_12)] | COVID-19 hospitalization | COVID-19 ICU admission | US | White | Hispanic | 4 | 4 | adjusted OR | 1 | income | 38 |  |  |
| Magesh, 2021[[12](#_ENREF_12)] | COVID-19 hospitalization | COVID-19 ICU admission | US | White | Hispanic | 4 | 4 | adjusted OR | 1 | Urban Core Opportunity Index (UOI) | 38 |  |  |
| Sze, 2020[[13](#_ENREF_13)] | COVID-19 hospitalization | COVID-19 ICU admission | US, UK | White | Hispanic | 3 | 3 | adjusted RR | ≥2 | at least 2 factors: age, sex. A prior set of confounders: age, sex, deprivation, obesity, and comorbidities | 38 |  |  |
| Agyemang, 2021[[11](#_ENREF_11)] | COVID-19 hospitalization | COVID-19 death | US | White | Black | 9 | 9 | adjusted OR | ≥ 3 | at least 3 factors are adjusted: Age, sex, comorbidities | 39 | select | largest number of studies |
| Magesh, 2021[[12](#_ENREF_12)] | COVID-19 hospitalization | COVID-19 death | US | White | Black | 7 | 7 | adjusted OR | 1 | income | 39 |  |  |
| Magesh, 2021[[12](#_ENREF_12)] | COVID-19 hospitalization | COVID-19 death | US | White | Black | 7 | 7 | adjusted OR | 1 | Area deprivation index (ADI) | 39 |  |  |
| Agyemang, 2021[[11](#_ENREF_11)] | COVID-19 hospitalization | COVID-19 death | US | White | Hispanic | 5 | 5 | adjusted OR | ≥ 3 | at least 3 factors are adjusted: Age, sex, comorbidities | 40 | select | largest number of studies |
| Magesh, 2021[[12](#_ENREF_12)] | COVID-19 hospitalization | COVID-19 death | US | White | Hispanic | 5 | 5 | adjusted OR | 1 | income | 40 |  |  |
| Magesh, 2021[[12](#_ENREF_12)] | COVID-19 hospitalization | COVID-19 death | US | White | Hispanic | 5 | 5 | adjusted OR | 1 | Area deprivation index (ADI) | 40 |  |  |
| Agyemang, 2021[[11](#_ENREF_11)] | COVID-19 hospitalization | COVID-19 death | US | White | Asian | 2 | 2 | adjusted OR | ≥ 3 | at least 3 factors are adjusted: Age, sex, comorbidities | 41 | select | largest number of studies + information of individual studies + largest number of adjusted factors |
| Magesh, 2021[[12](#_ENREF_12)] | COVID-19 hospitalization | COVID-19 death | US | White | Asian | 4 | 4 | adjusted OR | 1 | income | 41 |  |  |
| Magesh, 2021[[12](#_ENREF_12)] | COVID-19 hospitalization | COVID-19 death | US | White | Asian | 4 | 4 | adjusted OR | 1 | Area deprivation index (ADI) | 41 |  |  |
| Allotey, 2020[[17](#_ENREF_17)] | COVID-19 confirmed pregnancy | COVID-19 severity | Worldwide | White | White | 4 | 2 | unadjusted OR | 0 |  | 42 | select | unique association |
| Note: OR: Odds Ratio, RR: Risk Ratio, HR: Hazard Ratio, NA: Not available | | | | | | | | | | | | | |

## Supplementary Table 4: Criteria for Quality of Evidence Classification in Meta-analyses of Observational Studies

| **Strength of Evidence (Class)** | **Criteria** |
| --- | --- |
| Convincing (class 1) | • Number of participants >1000 • P < 10^-6^ • I^2^ < 50% • Largest component study reporting a nominal statistically significant result (P < .05) • 95% prediction interval excluding the null • No small-study effects |
| Highly suggestive (class 2) | • Number of participants >1000 • P < 10^-6^ • Largest study with a statistically significant effect (P < .05) |
| Suggestive (class 3) | • Number of participants >1000 • P < 10^-3^ |
| Weak (class 4) | P < .05 |
| Nonsignificant | P > .05 |

## Supplementary Table 5: All examined associations from included meta-analyses in the umbrella review

| **Author, Year** | **Population** | **Outcome** | **Setting** | **Ref.** | **Comp.** | **No. of studies in US** | **Effect measure** | **ES (95%CI)** | **p value** | **I^2^ (%)** | **No. cases >1000** | **Largest study (95% CI)** | **Prediction Interval** | **Small study effect** | **CE** | **AMSTAR 2** |
| --- | --- | --- | --- | --- | --- | --- | --- | --- | --- | --- | --- | --- | --- | --- | --- | --- |
| **Highly suggestive association** | | | | | | | | | | | | | | | | |
| Sze, 2020[[13](#_ENREF_13)] | General population | COVID-19 confirmed infection | US, UK | White | Black | 4 | adjusted RR | 2·08 (1·6–2·71) | 5·1x10^-8^ | 88·8 | Yes | 2·48(1·68–3·66) | 0·62–6·96 | 0·862 | II | Critical low |
| Agyemang, 2021[[11](#_ENREF_11)] | COVID-19 confirmed infection | COVID-19 hospitalization | US | White | Hispanic | 5 | adjusted OR | 2·08 (1·6–2·7) | 3·7x10^-8^ | 82·1 | Yes | 2·13(2·12–2·14) | 0·87–4·97 | 0·803 | II | Critical low |
| **Suggestive association** | | | | | | | | | | | | | | | | |
| Sze, 2020[[13](#_ENREF_13)] | General population | COVID-19 confirmed infection | US, UK | White | Hispanic | 2 | adjusted RR | 1·78 (1·37–2·31) | 1·3x10^-5^ | 0 | Yes | 1·68(1·18–2·33) | NA | NA | III | Critical low |
| Agyemang, 2021[[11](#_ENREF_11)] | COVID-19 confirmed infection | COVID-19 hospitalization | US | White | Black | 9 | adjusted OR | 1·53 (1·23–1·89) | 1·0x10^-4^ | 88 | Yes | 1·68(1·63–1·73) | 0·74–3·13 | 0·479 | III | Critical low |
| **Weak association** | | | | | | | | | | | | | | | | |
| Agyemang, 2021[[11](#_ENREF_11)] | General population | COVID-19 confirmed infection | US | White | Asian | 2 | adjusted OR | 1·74 (1·16–2·61) | 7·1x10^-3^ | 84·7 | NA | 2·16(1·68–2·79) | NA | NA | IV | Critical low |
| Agyemang, 2021[[11](#_ENREF_11)] | COVID-19 confirmed infection | COVID-19 hospitalization | US | White | Asian | 4 | adjusted OR | 1·59 (1·14–2·23) | 6·6x10^-3^ | 76·8 | NA | 1·29(0·97–1·72) | 0·37–6·89 | 0·503 | IV | Critical low |
| Mattey-Mora, 2021[[15](#_ENREF_15)] | COVID-19 confirmed infection | COVID-19 hospitalization | Mixed | non-Black | Black | 26 | unadjusted OR | 1·35 (1·05–1·74) | 0·018 | 99·2 | NA | 0·97(0·93–1) | 0·38–4·83 | 0·122 | IV | Low |
| Akbari, 2022[[16](#_ENREF_16)] | COVID-19 hospitalization | COVID-19 readmission hospital | US, UK | non-White | White | 6 | unadjusted OR | 1·47 (1·18–1·83) | 0·001 | 0 | No | 0·88(0·45–1·72) | 1·07–2·01 | 0·053 | IV | Critical low |
| **Non-significant association** | | | | | | | | | | | | | | | | |
| Mattey-Mora, 2021[[15](#_ENREF_15)] | COVID-19 confirmed infection | COVID-19 hospitalization | Mixed | non-White | White | 25 | unadjusted OR | 0·97 (0·85–1·1) | 0·616 | 96·8 | NA | 1·16(1·12–1·19) | 0·52–1·80 | 0·006 | NS | Low |
| Mattey-Mora, 2021[[15](#_ENREF_15)] | COVID-19 confirmed infection | COVID-19 hospitalization | Mixed | non-Hispanic | Hispanic | 22 | unadjusted OR | 1 (0·76–1·33) | 0·977 | 99·2 | NA | 2·38(2·32–2·43) | 0·26–3·86 | 0·009 | NS | Low |
| Raharja, 2021[[14](#_ENREF_14)] | COVID-19 confirmed infection | COVID-19 ICU admission | US, UK | White | Black | 3 | adjusted RR | 1·41 (0·86–2·33) | 0·176 | 93·5 | Yes | 1·95(1·62–2·39) | 0·003,698·43 | 0·673 | NS | Moderate |
| Raharja, 2021[[14](#_ENREF_14)] | COVID-19 confirmed infection | COVID-19 ICU admission | US, UK | White | Hispanic | 2 | adjusted RR | 0·93 (0·75–1·16) | 0·532 | 0 | No | 0·96(0·76–1·21) | NA | NA | NS | Moderate |
| Akbari, 2022[[16](#_ENREF_16)] | COVID-19 hospitalization | COVID-19 readmission ≤ 30 days (hospital/ED) | US, UK | non-White | White | 7 | unadjusted OR | 1·17 (0·81–1·68) | 0·405 | 56·1 | NA | 0·97(0·61–1·55) | 0·42–3·23 | 0·166 | NS | Critical low |
| Akbari, 2022[[16](#_ENREF_16)] | COVID-19 hospitalization | COVID-19 readmission ≤ 30 days (hospital/ED) | US, UK | non-Black | Black | 7 | unadjusted OR | 1·24 (0·78–1·95) | 0·361 | 65 | NA | 0·39(0·16–0·94) | 0·32–4·79 | 0·907 | NS | Critical low |
| Akbari, 2022[[16](#_ENREF_16)] | COVID-19 hospitalization | COVID-19 readmission ≤ 30 days (hospital/ED) | US, UK | non-Asian | Asian | 5 | unadjusted OR | 1·12 (0·8–1·56) | 0·517 | 0 | NA | 1·38(0·87–2·20) | 0·65–1·92 | 0·07 | NS | Critical low |
| Akbari, 2022[[16](#_ENREF_16)] | COVID-19 hospitalization | COVID-19 readmission ≤ 30 days (hospital/ED) | US, UK | non-Hispanic | Hispanic | 8 | unadjusted OR | 0·81 (0·58–1·15) | 0·236 | 57 | NA | 0·49(0·359–0·68) | 0·31–2·12 | 0·267 | NS | Critical low |
| Akbari, 2022[[16](#_ENREF_16)] | COVID-19 hospitalization | COVID-19 readmission > 30 days (hospital/ED) | US, UK | non-White | White | 3 | unadjusted OR | 1·36 (0·94–1·96) | 0·109 | 0 | NA | 1·48(0·92–2·38) | 0·12,14·97 | 0·153 | NS | Critical low |
| Akbari, 2022[[16](#_ENREF_16)] | COVID-19 hospitalization | COVID-19 readmission > 30 days (hospital/ED) | US, UK | non-Black | Black | 3 | unadjusted OR | 0·76 (0·5–1·15) | 0·197 | 0 | NA | 0·78(0·50–1·23) | 0·05,11·44 | 0·973 | NS | Critical low |
| Akbari, 2022[[16](#_ENREF_16)] | COVID-19 hospitalization | COVID-19 readmission > 30 days (hospital/ED) | US, UK | non-Asian | Asian | 3 | unadjusted OR | 0·49 (0·17–1·38) | 0·175 | 0 | NA | 0·18(0·02–1·38) | 0·001,416·07 | 0·779 | NS | Critical low |
| Akbari, 2022[[16](#_ENREF_16)] | COVID-19 hospitalization | COVID-19 readmission > 30 days (hospital/ED) | US, UK | non-Hispanic | Hispanic | 2 | unadjusted OR | 0·78 (0·41–1·48) | 0·444 | 0 | NA | 0·77(0·35–1·73) | NA | NA | NS | Critical low |
| Akbari, 2022[[16](#_ENREF_16)] | COVID-19 hospitalization | COVID-19 readmission hospital | US, UK | non-Black | Black | 6 | unadjusted OR | 0·83 (0·56–1·24) | 0·366 | 51·4 | NA | 0·78(0·50–1·23) | 0·28–2·52 | 0·192 | NS | Critical low |
| Akbari, 2022[[16](#_ENREF_16)] | COVID-19 hospitalization | COVID-19 readmission hospital | US, UK | non-Asian | Asian | 5 | unadjusted OR | 0·91 (0·54–1·53) | 0·714 | 33·1 | NA | 1·38(0·87–2·20) | 0·23–3·59 | 0·02 | NS | Critical low |
| Akbari, 2022[[16](#_ENREF_16)] | COVID-19 hospitalization | COVID-19 readmission hospital | US, UK | non-Hispanic | Hispanic | 7 | unadjusted OR | 0·77 (0·54–1·11) | 0·164 | 56 | NA | 0·49(0·36–0·68) | 0·28–2·16 | 0·085 | NS | Critical low |
| Akbari, 2022[[16](#_ENREF_16)] | COVID-19 hospitalization | COVID-19 readmission hospital/ED | US, UK | non-White | White | 10 | unadjusted OR | 1·23 (0·96–1·59) | 0·107 | 36·7 | NA | 0·97(0·61–1·55) | 0·66–2·31 | 0·088 | NS | Critical low |
| Akbari, 2022[[16](#_ENREF_16)] | COVID-19 hospitalization | COVID-19 readmission hospital/ED | US, UK | non-Black | Black | 10 | unadjusted OR | 1·09 (0·76–1·59) | 0·635 | 58·4 | NA | 0·78(0·50–1·23) | 0·38–3·17 | 0·926 | NS | Critical low |
| Akbari, 2022[[16](#_ENREF_16)] | COVID-19 hospitalization | COVID-19 readmission hospital/ED | US, UK | non-Asian | Asian | 8 | unadjusted OR | 1·03 (0·75–1·42) | 0·840 | 0 | NA | 1·38(0·87–2·20) | 0·69–1·54 | 0·011 | NS | Critical low |
| Akbari, 2022[[16](#_ENREF_16)] | COVID-19 hospitalization | COVID-19 readmission hospital/ED | US, UK | non-Hispanic | Hispanic | 10 | unadjusted OR | 0·8 (0·6–1·06) | 0·124 | 44·9 | NA | 0·49(0·36–0·68) | 0·38–1·69 | 0·223 | NS | Critical low |
| Raharja, 2021[[14](#_ENREF_14)] | COVID-19 confirmed infection | acute kidney injuries | US, UK | White | Black | 2 | adjusted RR | 1·6 (0·88–2·91) | 0·123 | 94·7 | Yes | 2·18(1·75–2·72) | NA | NA | NS | Moderate |
| Raharja, 2021[[14](#_ENREF_14)] | COVID-19 confirmed infection | invasive mechanical ventilation | US, UK | White | Black | 3 | adjusted RR | 1·23 (0·61–2·5) | 0·567 | 90·8 | No | 2·36(1·72–3·25) | 0·000–8565·09 | 0·376 | NS | Moderate |
| Raharja, 2021[[14](#_ENREF_14)] | COVID-19 confirmed infection | invasive mechanical ventilation | US, UK | White | Hispanic | 2 | adjusted RR | 1·01 (0·84–1·21) | 0·933 | 0 | No | 0·85(0·51–1·43) | NA | NA | NS | Moderate |
| Sze, 2020[[13](#_ENREF_13)] | COVID-19 confirmed infection | COVID-19 death | US, UK | White | Black | 15 | adjusted HR/RR | 1·03 (0·9–1·17) | 0·695 | 39·5 | NA | 0·93(0·80–1·09) | 0·73–1·44 | 0·924 | NS | Critical low |
| Sze, 2020[[13](#_ENREF_13)] | COVID-19 confirmed infection | COVID-19 death | US, UK | White | Asian | 3 | adjusted HR/RR | 0·96 (0·78–1·17) | 0·676 | 0 |  | 0·97(0·76–1·25) | 0·26–3·560 | 0·348 | NS | Critical low |
| Agyemang, 2021[[11](#_ENREF_11)] | COVID-19 hospitalization | COVID-19 severity | US | White | Black | 3 | adjusted OR | 1·54 (0·82–2·88) | 0·177 | 76·7 | NA | 1·31(0·97–1·77) | 0·001–2374·52 | 0·829 | NS | Critical low |
| Agyemang, 2021[[11](#_ENREF_11)] | COVID-19 hospitalization | COVID-19 severity | US | White | Hispanic | 2 | adjusted OR | 1·83 (0·9–3·71) | 0·095 | 57·7 | NA | 1·34(0·79–2·28) | NA | NA | NS | Critical low |
| Agyemang, 2021[[11](#_ENREF_11)] | COVID-19 hospitalization | COVID-19 severity | US | White | Asian | 3 | unadjusted OR | 3·11 (1·84–5·24) | 2·2x10^-5^ | 3·3 | No | 2·13(0·82–5·55) | 0·08–114·51 | 0·276 | IV | Critical low |
| Sze, 2020[[13](#_ENREF_13)] | COVID-19 hospitalization | COVID-19 ICU admission | US, UK | White | Black | 3 | adjusted RR | 1·14 (0·73–1·8) | 0·566 | 67·7 | NA | 1·01(0·89–1·15) | 0·01–175·07 | 0·64 | NS | Critical low |
| Magesh, 2021[[12](#_ENREF_12)] | COVID-19 hospitalization | COVID-19 ICU admission | US | White | Asian | 3 | adjusted OR | 2·09 (1·59–2·76) | NA | NA | NA | NA | NA | NA | IV | Critical low |
| Agyemang, 2021[[11](#_ENREF_11)] | COVID-19 hospitalization | COVID-19 ICU admission | US | White | Hispanic | 3 | adjusted OR | 1·22 (0·79–1·88) | 0·378 | 49·4 | NA | 0·96(0·76–1·21) | 0·01–104·07 | 0·201 | NS | Critical low |
| Agyemang, 2021[[11](#_ENREF_11)] | COVID-19 hospitalization | COVID-19 death | US | White | Black | 9 | adjusted OR | 0·98 (0·88–1·1) | 0·733 | 35·2 | NA | 1·03(0·98–1·1) | 0·76–1·26 | 0·355 | NS | Critical low |
| Agyemang, 2021[[11](#_ENREF_11)] | COVID-19 hospitalization | COVID-19 death | US | White | Hispanic | 5 | adjusted OR | 0·9 (0·61–1·31) | 0·567 | 97·7 | NA | 1·14(1·11–1·15) | 0·22–3·57 | 0·401 | NS | Critical low |
| Agyemang, 2021[[11](#_ENREF_11)] | COVID-19 hospitalization | COVID-19 death | US | White | Asian | 2 | adjusted OR | 1·1 (0·72–1·68) | 0·671 | 62·8 | NA | 0·94(0·83–1·08) | NA | NA | NS | Critical low |
| Allotey, 2020[[17](#_ENREF_17)] | COVID-19 confirmed pregnancy | COVID-19 severity | Mixed | White | Non-White | 2 | unadjusted OR | 0·53 (0·24–1·17) | 0·113 | 0 | No | 0·45(0·19–1·06) | NA | NA | NS | Critical low |
| Note: OR: Odds Ratio, RR: Risk Ratio, HR: Hazard Ratio, NA: Not available, NS: Non-significant, Ref.: Reference, Comp.: Comparator, ES: Effect Size, CI: Confidence Interval, CE: Class of Evidence | | | | | | | | | | | | | | | | |

## Supplementary Table 6: Descriptive characteristics of included meta-analysis

| **Author, Year** | **Period of study conducted** | **Population** | **Outcome** | **Setting** | **Reference** | **Comparator** | **No. of studies in US** | **Effect measure** | **No. of adjusted factors** | **Adjusted factors** |
| --- | --- | --- | --- | --- | --- | --- | --- | --- | --- | --- |
| Sze, 2020[[13](#_ENREF_13)] | April,2020 - August, 2020 | General population | COVID-19 confirmed infection | US, UK | White | Black | 4 | adjusted RR | ≥ 2 | at least 2 factors: age, sex. A prior set of confounders: age, sex, deprivation, obesity, and comorbidities |
| Agyemang, 2021[[11](#_ENREF_11)] | Jan,2020 - August, 2020 | General population | COVID-19 confirmed infection | US | White | Asian | 2 | adjusted OR | ≥ 3 | at least 3 factors are adjusted: Age, sex, comorbidities |
| Sze, 2020[[13](#_ENREF_13)] | April,2020 - August, 2020 | General population | COVID-19 confirmed infection | US, UK | White | Hispanic | 2 | adjusted RR | ≥2 | at least 2 factors: age, sex. A prior set of confounders: age, sex, deprivation, obesity, and comorbidities |
| Agyemang, 2021[[11](#_ENREF_11)] | Jan,2020 - August, 2020 | COVID-19 confirmed infection | COVID-19 hospitalization | US | White | Black | 9 | adjusted OR | ≥ 3 | at least 3 factors are adjusted: Age, sex, comorbidities |
| Agyemang, 2021[[11](#_ENREF_11)] | Jan,2020 - August, 2020 | COVID-19 confirmed infection | COVID-19 hospitalization | US | White | Asian | 4 | adjusted OR | ≥ 3 | at least 3 factors are adjusted: Age, sex, comorbidities |
| Agyemang, 2021[[11](#_ENREF_11)] | Jan,2020 - August, 2020 | COVID-19 confirmed infection | COVID-19 hospitalization | US | White | Hispanic | 5 | adjusted OR | ≥ 3 | at least 3 factors are adjusted: Age, sex, comorbidities |
| Mattey-Mora, 2021[[15](#_ENREF_15)] | Feb 2020 - June, 2020 | COVID-19 confirmed infection | COVID-19 hospitalization | Mixed | non-White | White | 25 | unadjusted OR | 0 | Not adjusted |
| Mattey-Mora, 2021[[15](#_ENREF_15)] | Feb 2020 - June, 2020 | COVID-19 confirmed infection | COVID-19 hospitalization | Mixed | non-Black | Black | 26 | unadjusted OR | 0 | Not adjusted |
| Mattey-Mora, 2021[[15](#_ENREF_15)] | Feb 2020 - June, 2020 | COVID-19 confirmed infection | COVID-19 hospitalization | Mixed | non-Hispanic | Hispanic | 22 | unadjusted OR | 0 | Not adjusted |
| Raharja, 2021[[14](#_ENREF_14)] | 2020 | COVID-19 confirmed infection | COVID-19 ICU admission | US, UK | White | Black | 3 | adjusted RR | 3 | age, sex, comorbidities |
| Raharja, 2021[[14](#_ENREF_14)] | 2020 | COVID-19 confirmed infection | COVID-19 ICU admission | US, UK | White | Hispanic | 2 | adjusted RR | 3 | age, sex, comorbidities |
| Akbari, 2022[[16](#_ENREF_16)] | 2020 | COVID-19 hospitalization | COVID-19 readmission ≤ 30 days (hospital/ED) | US, UK | non-White | White | 7 | unadjusted OR | 0 | Not adjusted |
| Akbari, 2022[[16](#_ENREF_16)] | 2020 | COVID-19 hospitalization | COVID-19 readmission ≤ 30 days (hospital/ED) | US, UK | non-Black | Black | 7 | unadjusted OR | 0 | Not adjusted |
| Akbari, 2022[[16](#_ENREF_16)] | 2020 | COVID-19 hospitalization | COVID-19 readmission ≤ 30 days (hospital/ED) | US, UK | non-Asian | Asian | 5 | unadjusted OR | 0 | Not adjusted |
| Akbari, 2022[[16](#_ENREF_16)] | 2020 | COVID-19 hospitalization | COVID-19 readmission ≤ 30 days (hospital/ED) | US, UK | non-Hispanic | Hispanic | 8 | unadjusted OR | 0 | Not adjusted |
| Akbari, 2022[[16](#_ENREF_16)] | 2020 | COVID-19 hospitalization | COVID-19 readmission > 30 days (hospital/ED) | US, UK | non-White | White | 3 | unadjusted OR | 0 | Not adjusted |
| Akbari, 2022[[16](#_ENREF_16)] | 2020 | COVID-19 hospitalization | COVID-19 readmission > 30 days (hospital/ED) | US, UK | non-Black | Black | 3 | unadjusted OR | 0 | Not adjusted |
| Akbari, 2022[[16](#_ENREF_16)] | 2020 | COVID-19 hospitalization | COVID-19 readmission > 30 days (hospital/ED) | US, UK | non-Asian | Asian | 3 | unadjusted OR | 0 | Not adjusted |
| Akbari, 2022[[16](#_ENREF_16)] | 2020 | COVID-19 hospitalization | COVID-19 readmission > 30 days (hospital/ED) | US, UK | non-Hispanic | Hispanic | 2 | unadjusted OR | 0 | Not adjusted |
| Akbari, 2022[[16](#_ENREF_16)] | 2020 | COVID-19 hospitalization | COVID-19 readmission hospital | US, UK | non-White | White | 6 | unadjusted OR | 0 | Not adjusted |
| Akbari, 2022[[16](#_ENREF_16)] | 2020 | COVID-19 hospitalization | COVID-19 readmission hospital | US, UK | non-Black | Black | 6 | unadjusted OR | 0 | Not adjusted |
| Akbari, 2022[[16](#_ENREF_16)] | 2020 | COVID-19 hospitalization | COVID-19 readmission hospital | US, UK | non-Asian | Asian | 5 | unadjusted OR | 0 | Not adjusted |
| Akbari, 2022[[16](#_ENREF_16)] | 2020 | COVID-19 hospitalization | COVID-19 readmission hospital | US, UK | non-Hispanic | Hispanic | 7 | unadjusted OR | 0 | Not adjusted |
| Akbari, 2022[[16](#_ENREF_16)] | 2020 | COVID-19 hospitalization | COVID-19 readmission hospital/ED | US, UK | non-White | White | 10 | unadjusted OR | 0 | Not adjusted |
| Akbari, 2022[[16](#_ENREF_16)] | 2020 | COVID-19 hospitalization | COVID-19 readmission hospital/ED | US, UK | non-Black | Black | 10 | unadjusted OR | 0 | Not adjusted |
| Akbari, 2022[[16](#_ENREF_16)] | 2020 | COVID-19 hospitalization | COVID-19 readmission hospital/ED | US, UK | non-Asian | Asian | 8 | unadjusted OR | 0 | Not adjusted |
| Akbari, 2022[[16](#_ENREF_16)] | 2020 | COVID-19 hospitalization | COVID-19 readmission hospital/ED | US, UK | non-Hispanic | Hispanic | 10 | unadjusted OR | 0 | Not adjusted |
| Raharja, 2021[[14](#_ENREF_14)] | 2020 | COVID-19 confirmed infection | acute kidney injuries | US, UK | White | Black | 2 | adjusted RR | 3 | age, sex, comorbidities |
| Raharja, 2021[[14](#_ENREF_14)] | 2020 | COVID-19 confirmed infection | invasive mechanical ventilation | US, UK | White | Black | 3 | adjusted RR | 3 | age, sex, comorbidities |
| Raharja, 2021[[14](#_ENREF_14)] | 2020 | COVID-19 confirmed infection | invasive mechanical ventilation | US, UK | White | Hispanic | 2 | adjusted RR | 3 | age, sex, comorbidities |
| Sze, 2020[[13](#_ENREF_13)] | April,2020 - August, 2020 | COVID-19 confirmed infection | COVID-19 death | US, UK | White | Black | 15 | adjusted HR/RR | ≥2 | at least 2 factors: age, sex. A prior set of confounders: age, sex, deprivation, obesity, and comorbidities |
| Sze, 2020[[13](#_ENREF_13)] | April,2020 - August, 2020 | COVID-19 confirmed infection | COVID-19 death | US, UK | White | Asian | 3 | adjusted HR/RR | ≥2 | at least 2 factors: age, sex. A prior set of confounders: age, sex, deprivation, obesity, and comorbidities |
| Agyemang, 2021[[11](#_ENREF_11)] | Jan,2020 - August, 2020 | COVID-19 hospitalization | COVID-19 severity | US | White | Black | 3 | adjusted OR | ≥ 3 | at least 3 factors are adjusted: Age, sex, comorbidities |
| Agyemang, 2021[[11](#_ENREF_11)] | Jan,2020 - August, 2020 | COVID-19 hospitalization | COVID-19 severity | US | White | Hispanic | 2 | adjusted OR | ≥ 3 | at least 3 factors are adjusted: Age, sex, comorbidities |
| Agyemang, 2021[[11](#_ENREF_11)] | Jan,2020 - August, 2020 | COVID-19 hospitalization | COVID-19 severity | US | White | Asian | 3 | unadjusted OR | 0 | Not adjusted |
| Sze, 2020[[13](#_ENREF_13)] | April,2020 - August, 2020 | COVID-19 hospitalization | COVID-19 ICU admission | US, UK | White | Black | 3 | adjusted RR | ≥2 | at least 2 factors: age, sex. A prior set of confounders: age, sex, deprivation, obesity, and comorbidities |
| Magesh, 2021[[12](#_ENREF_12)] | 2020 | COVID-19 hospitalization | COVID-19 ICU admission | US | White | Asian | 3 | adjusted OR | 1 | age |
| Agyemang, 2021[[11](#_ENREF_11)] | Jan,2020 - August, 2020 | COVID-19 hospitalization | COVID-19 ICU admission | US | White | Hispanic | 3 | adjusted OR | ≥ 3 | at least 3 factors are adjusted: Age, sex, comorbidities |
| Agyemang, 2021[[11](#_ENREF_11)] | Jan,2020 - August, 2020 | COVID-19 hospitalization | COVID-19 death | US | White | Black | 9 | adjusted OR | ≥ 3 | at least 3 factors are adjusted: Age, sex, comorbidities |
| Agyemang, 2021[[11](#_ENREF_11)] | Jan,2020 - August, 2020 | COVID-19 hospitalization | COVID-19 death | US | White | Hispanic | 5 | adjusted OR | ≥ 3 | at least 3 factors are adjusted: Age, sex, comorbidities |
| Agyemang, 2021[[11](#_ENREF_11)] | Jan,2020 - August, 2020 | COVID-19 hospitalization | COVID-19 death | US | White | Asian | 2 | adjusted OR | ≥ 3 | at least 3 factors are adjusted: Age, sex, comorbidities |
| Allotey, 2020[[17](#_ENREF_17)] | 2020 | COVID-19 confirmed pregnancy | COVID-19 severity | Mixed | White | Non-White | 2 | unadjusted OR | 0 | Not adjusted |
| Note: OR: Odds Ratio, RR: Risk Ratio, HR: Hazard Ratio | | | | | | | | | | |

## Supplementary Table 7: Sensitivity analysis of associations initially graded as highly suggestive and suggestive evidence

| **Author, Year** | **Population** | **Outcome** | **Ref.** | **Comp.** | **Effect measure** | **Primary analysis** | | | **Sensitivity analysis: Excluding low quality of studies** | | | **Sensitivity analysis: Excluding small size studies (less than 25^th^ percentile)** | | |
| --- | --- | --- | --- | --- | --- | --- | --- | --- | --- | --- | --- | --- | --- | --- |
|  |  |  |  |  |  | **No. of studies** | **ES (95%CI)** | **CE** | **No. of studies** | **ES (95%CI)** | **CE** | **No. of studies** | **ES (95%CI)** | **CE** |
| Sze, 2020[[13](#_ENREF_13)] | General population | COVID-19 confirmed infection | White | Black | RR | 4 | 2·08 (1·60–2·71) | II | 4 | 2·08 (1·60–2·71) | II | 3 | ^a^1·90 (1·43–2·52) | III |
| Sze, 2020[[13](#_ENREF_13)] | General population | COVID-19 confirmed infection | White | Hispanic | RR | 2 | 1·78 (1·37–2·31) | III | 2 | 1·78 (1·37–2·31) | III | NA | NA | NA |
| Agyemang, 2021[[11](#_ENREF_11)] | COVID-19 confirmed infection | COVID-19 hospitalization | White | Black | OR | 9 | 1·53 (1·23–1·89) | III | NA | NA | NA | 7 | ^b^1·38 (1·09–1·74) | IV |
| Agyemang, 2021[[11](#_ENREF_11)] | COVID-19 confirmed infection | COVID-19 hospitalization | White | Hispanic | OR | 5 | 2·08 (1·60–2·70) | II | NA | NA | NA | 4 | ^c^2·11 (1·57–2·83) | II |
| a: Heterogeneity I^2^_:_ 82.1%, No. of case >1000: Yes, Largest study (95% CI): 2·48(1·68–3·66), PrI: 0·07, 52·37, Egger’s test: 0·862, AMSTAR 2:Critical low  b: Heterogeneity I^2^_:_ 90.0%, No. of case >1000: Yes, Largest study (95% CI): 1·20(0·68–2·12), PrI: 0·62, 3·03, Egger’s test: 0·479, AMSTAR 2:Critical low  c: Heterogeneity I^2^_:_ 86.5%, No. of case >1000: Yes, Largest study (95% CI): 2·13(2·12–2·14,), PrI: 0·59, 7·52, Egger’s test: 0·803, AMSTAR 2:Critical low  Note: Note: OR: Odds Ratio, RR: Risk Ratio, HR: Hazard Ratio, NA: Not available, NS: Non-significant, Ref.: Reference, Comp.: Comparator, ES: Effect Size, CI: Confidence Interval, CE: Class of Evidence, PrI: Prediction Interval | | | | | | | | | | | | | | |

## References

**1.** Belbasis L, Bellou V, Evangelou E, Ioannidis JPA, Tzoulaki I. Environmental risk factors and multiple sclerosis: an umbrella review of systematic reviews and meta-analyses. *The Lancet Neurology* **14** (2015).263-73. doi: 10.1016/S1474-4422(14)70267-4

**2.** Brabaharan S, Veettil SK, Kaiser JE, Raja Rao VR, Wattanayingcharoenchai R, Maharajan M, et al. Association of Hormonal Contraceptive Use With Adverse Health Outcomes: An Umbrella Review of Meta-analyses of Randomized Clinical Trials and Cohort Studies. *JAMA Netw Open* **5** (2022).e2143730. doi: 10.1001/jamanetworkopen.2021.43730

**3.** Dragioti E, Solmi M, Favaro A, Fusar-Poli P, Dazzan P, Thompson T, et al. Association of Antidepressant Use With Adverse Health Outcomes: A Systematic Umbrella Review. *JAMA Psychiatry* **76** (2019).1241-55. doi: 10.1001/jamapsychiatry.2019.2859

**4.** Veettil SK, Wong TY, Loo YS, Playdon MC, Lai NM, Giovannucci EL, et al. Role of Diet in Colorectal Cancer Incidence: Umbrella Review of Meta-analyses of Prospective Observational Studies. *JAMA Network Open* **4** (2021).e2037341-e. doi: 10.1001/jamanetworkopen.2020.37341

**5.** Shea BJ, Reeves BC, Wells G, Thuku M, Hamel C, Moran J, et al. AMSTAR 2: a critical appraisal tool for systematic reviews that include randomised or non-randomised studies of healthcare interventions, or both. *Bmj* **358** (2017).j4008. doi: 10.1136/bmj.j4008

**6.** He Y, Li X, Gasevic D, Brunt E, McLachlan F, Millenson M, et al. Statins and Multiple Noncardiovascular Outcomes: Umbrella Review of Meta-analyses of Observational Studies and Randomized Controlled Trials. *Ann Intern Med* **169** (2018).543-53. doi: 10.7326/m18-0808

**7.** Alhumaid S, Al Mutair A, Al Alawi Z, Rabaan AA, Alomari MA, Al Salman SA, et al. Diabetic ketoacidosis in patients with SARS-CoV-2: a systematic review and meta-analysis. *Diabetology and Metabolic Syndrome* **13** (2021). doi: 10.1186/s13098-021-00740-6

**8.** Bhakta S, Erben Y, Sanghavi D, Fortich S, Li Y, Hasan MM, et al. A systematic review and meta-analysis of racial disparities in deep vein thrombosis and pulmonary embolism events in patients hospitalized with coronavirus disease 2019. *Journal of Vascular Surgery: Venous and Lymphatic Disorders* (2022). doi: 10.1016/j.jvsv.2022.03.003

**9.** Khan DSA, Hamid LR, Ali A, Salam RA, Zuberi N, Lassi ZS, et al. Differences in pregnancy and perinatal outcomes among symptomatic versus asymptomatic COVID-19-infected pregnant women: a systematic review and meta-analysis. *BMC Pregnancy and Childbirth* **21** (2021). doi: 10.1186/s12884-021-04250-1

**10.** Mude W, Oguoma VM, Nyanhanda T, Mwanri L, Njue C. Racial disparities in COVID-19 pandemic cases, hospitalisations, and deaths: A systematic review and meta-analysis. *J Glob Health* **11** (2021).05015. doi: 10.7189/jogh.11.05015

**11.** Agyemang C, Richters A, Jolani S, Hendriks S, Zalpuri S, Yu E, et al. Ethnic minority status as social determinant for COVID-19 infection, hospitalisation, severity, ICU admission and deaths in the early phase of the pandemic: a meta-analysis. *BMJ Glob Health* **6** (2021). doi: 10.1136/bmjgh-2021-007433

**12.** Magesh S, John D, Li WT, Li Y, Mattingly-App A, Jain S, et al. Disparities in COVID-19 Outcomes by Race, Ethnicity, and Socioeconomic Status: A Systematic-Review and Meta-analysis. *JAMA Netw Open* **4** (2021).e2134147. doi: 10.1001/jamanetworkopen.2021.34147

**13.** Sze S, Pan D, Nevill CR, Gray LJ, Martin CA, Nazareth J, et al. Ethnicity and clinical outcomes in COVID-19: A systematic review and meta-analysis. *EClinicalMedicine* **29** (2020).100630. doi: 10.1016/j.eclinm.2020.100630

**14.** Raharja A, Tamara A, Kok LT. Association Between Ethnicity and Severe COVID-19 Disease: a Systematic Review and Meta-analysis. *J Racial Ethn Health Disparities* **8** (2021).1563-72. doi: 10.1007/s40615-020-00921-5

**15.** Mattey-Mora PP, Begle CA, Owusu CK, Chen C, Parker MA. Hospitalised versus outpatient COVID-19 patients' background characteristics and comorbidities: A systematic review and meta-analysis. *Reviews in Medical Virology* **32** (2022).e2306. doi: 10.1002/rmv.2306

**16.** Akbari A, Fathabadi A, Razmi M, Zarifian A, Amiri M, Ghodsi A, et al. Characteristics, risk factors, and outcomes associated with readmission in COVID-19 patients: A systematic review and meta-analysis. *American Journal of Emergency Medicine* **52** (2022).166-73. doi: 10.1016/j.ajem.2021.12.012

**17.** Allotey J, Stallings E, Bonet M, Yap M, Chatterjee S, Kew T, et al. Clinical manifestations, risk factors, and maternal and perinatal outcomes of coronavirus disease 2019 in pregnancy: living systematic review and meta-analysis. *Bmj* **370** (2020).m3320. doi: 10.1136/bmj.m3320
